# Coordinated Activity of Photosystem I and Photosystem II in *Selaginella martensii* (Lycopodiophyta) Across Light Gradients

**DOI:** 10.1111/ppl.70857

**Published:** 2026-04-04

**Authors:** Lorenzo Ferroni, Marek Živčak, Andrea Colpo, Stefania Simonetto, Costanza Baldisserotto, Simonetta Pancaldi, Marian Brestič

**Affiliations:** ^1^ Department of Environmental and Prevention Sciences University of Ferrara Ferrara Italy; ^2^ Institute of Plant and Environmental Sciences, Faculty of Agrobiology and Food Resources Slovak University of Agriculture Nitra Slovakia

**Keywords:** chlorophyll a fluorescence, photosynthetic electron transport, *Selaginella martensii*, shade acclimation, ultrastructure

## Abstract

*Selaginella martensii* is a living member of lycophytes, an early divergent group of vascular plants. This shade‐adapted species can acclimate to various light regimes, from deep shade to high light, with a gain in carboxylation capacity accompanied by modulations in thylakoid composition, including increased relative amount of photosystem I (PSI) compared to photosystem II (PSII). It was hypothesized that, as in angiosperms, the photosynthetic acclimation of *S. martensii* to high light may lead to increased cyclic electron flow around PSI (CEF), with changes in excitation distribution between PSI and PSII and with modulations of electron carrier pools. After long‐term acclimation to deep shade (LL), intermediate shade (ML) and high light (HL) regimes, plants were compared using chlorophyll *a* fluorescence, P700 oxidation, electrochromic bandshift, and transmission electron microscopy of the thylakoid system. As expected, a 75% increase in CEF occurred from LL to HL plants, facilitated by a preferential energy distribution to PSI. However, some unique characteristics distinguish *S. martensii* from common models of long‐term thylakoid photoregulation. The pools of electron transporters did not increase linearly with the electron fluxes from LL to HL plants; rather, the enhancement of electron transport depended on unique combinations of capacity and mobility of the electron carriers, the latter determined by the extent of thylakoid shrinkage. Moreover, especially in shade‐acclimated plants, the fraction of oxidized PSI exceeded that of reduced Q_A_, therefore keeping PSI under a persistent shortage of electrons already at low light intensities.

## 1 Introduction

After the outstanding success of lycophytes up to the Carboniferous period, the subsequent thriving of euphyllophytes, including particularly angiosperms (Benton et al. 2022), was accompanied by the extinction of the major part of the former and relegation of the remaining ones mostly to humid shaded environments of rain forests (Spencer et al. 2021). During a million years of evolution, many *Selaginella* species, the largest group of living lycophytes, developed striking adaptations to living under the shade of other plants, including a unique condition of monoplastidy in the leaf upper epidermis (Sheue et al. 2007; Liu et al. 2020). Inside the chloroplast, a very large antenna system puts emphasis on the light harvesting; accordingly, a quite high degree of appression characterizes the thylakoid system (Sheue et al. 2007; Colpo et al. 2023, 2024). Because the Calvin–Benson–Bassham cycle is a weak electron sink in *Selaginella* (Ferroni, Brestič, et al. 2021), the photosynthetic electron chain from photosystem II (PSII) to photosystem I (PSI) end acceptors is promptly saturated already at relatively low irradiance (Ferroni et al. 2014; Ferroni, Brestič, et al. 2021), potentially leading to overexcitation of PSII and overreduction of PSI. Accumulation of reduced electron carriers at the PSI donor side could expose PSI to photodamage, a lethal occurrence (Sonoike 2011; Lima‐Melo et al. 2021; Shimakawa et al. 2024). Nevertheless, in vascular plants, numerous regulatory mechanisms of the photosynthetic membrane ultimately converge toward the preservation of PSI integrity, preventing PSI overreduction and, conversely, promoting the safe accumulation of oxidized P700 (Grieco et al. 2012; Shimakawa and Miyake 2018; Barbato et al. 2020). Alleviation of the electron pressure on PSI can be obtained by lowering the influx of electrons into the chain and by increasing the electron efflux from PSI (Shimakawa and Miyake 2018).

The thermal dissipation of excess absorbed energy, known as the non‐photochemical quenching of chlorophyll fluorescence (NPQ) and specifically its major “high energy” component qE, is considered a primary mechanism to down‐regulate PSII photochemical activity (Ruban 2016). Induced by the trans‐thylakoidal ΔpH, NPQ has generally been assigned a direct photoprotective role of PSII and, by decreasing the electron inflow into the transport chain, an indirect protective role of PSI (Demmig‐Adams et al. 2014). However, NPQ most probably downregulates both photosystems to a similar extent, allowing the control of PSI and PSII excitation balance through the light‐harvesting complex II (LHCII) shared between them (Grieco et al. 2015). While NPQ limits the overreduction of the plastoquinone (PQ) pool, it seems not required for the accumulation of oxidized P700 (Tikkanen et al. 2015; Barbato et al. 2020). The facilitation of PSI oxidation occurs instead through mechanisms that divert the photosynthetic electrons produced in excess of the capacity for utilization by the Calvin–Benson–Bassham cycle. The primary donor‐side regulator of PSI oxidation state is cytochrome *b*_*6*_ *f* (cyt*b*_*6*_ *f*), which is responsible for the “photosynthetic control” that restricts the linear electron flow from PSII to PSI (LEF) and, at the same time, is engaged in sustaining the cyclic electron flow around PSI (CEF; Malone et al. 2021; Degen and Johnson 2024). With CEF, electrons are shuttled back to the PQ pool and again to PSI (Nawrocki et al. 2019), and such an electron recirculation is accompanied by the building up of the ΔpH to enhance the synthesis of ATP, but also to further induce NPQ (Munekage et al. 2001, 2002; Suorsa et al. 2012). Under certain environmental conditions, such as chilling stress or light intensity fluctuations, PSII photoinhibition is another protective strategy to preserve PSI against overreduction and photodamage (Takeuchi et al. 2025). Downstream of PSI, acceptor‐side systems can remove electrons from the electron transport chain, such as photorespiration (Timm et al. 2024) and additional pathways that consume electrons using sinks alternative to CO_2_ (Alric and Johnson 2017), particularly O_2_ through the water–water cycle and the plastid terminal oxidase (Miyake 2010; Sun et al. 2020; Messant et al. 2024) and, in non‐angiosperms—*Selaginella* included –, flavodiiron proteins (FLVs; Allahverdiyeva et al. 2015; Ilík et al. 2017; Bag et al. 2023).

Despite its evolutionary marked shade character, *S. martensii* has a singular ability to effectively long‐term acclimate to contrasting natural light gradients (Ferroni et al. 2016; Ferroni, Brestič, et al. 2021; Colpo et al. 2022). From deep shade to full sunlight, profound changes occur in the photosynthetic membrane of *S. martensii* , particularly an increase in the PSI/PSII ratio, accompanied by a higher relative abundance of cyt*b*_*6*_ *f* and ATP synthase. Contrastingly, no major modulation affects the relative amount of LHCII or the NPQ‐related PsbS protein (Ferroni et al. 2016). Accordingly, in all conditions, *S. martensii* maintains a fast and intense induction of NPQ (Ferroni et al. 2014; Ferroni, Colpo, et al. 2021), although the effectiveness of PSII photoprotection increases from deep shade to high light‐cultivated plants (Colpo et al. 2022). Likewise, each condition of long‐term acclimation is permissive to the accumulation of high levels of oxidized PSI centers, which, at saturation, approach dissipation of approx. 80% of the light energy reaching the photosystem (Ferroni et al. 2016). Therefore, along with PSII and PSI de‐excitation by NPQ, an effective parallel regulation must operate on the photosynthetic electron flows to prevent PSI overreduction. For example, the use of alternative electron sinks was shown to increase in parallel with the gain in carboxylation capacity from deep shade to high light‐acclimated plants (Ferroni, Brestič, et al. 2021). The main aim of this study was to verify whether CEF is similarly upregulated. After having obtained information on the functional organization of PSII antenna and on photosynthetic electron carriers by fast chlorophyll *a* fluorescence, we induced photosynthesis to a saturated quasi‐steady state and subsequently triggered light curves of chlorophyll *a* fluorescence and P700 absorption to calculate CEF. Beside the expected variations in CEF, we highlight that *S. martensii* is perfectly protected against overreduction of PSI acceptor side at high light intensities, even when the plant has been long‐term acclimated to deep shade.

## 2 Materials and Methods

### 2.1 Plant Material and Growth Conditions

Plants of *Selaginella martensii* Spring (Lycopodiophyta, Selaginellaceae) were routinely grown in pots in a warm, humid greenhouse (25°C–30°C) at the Botanical Garden of the University of Ferrara under natural photoperiod (N 44°50′30″, E 11°37′22″). The long‐term light acclimation protocol was done as previously reported (Ferroni et al. 2016; Ferroni, Brestič, et al. 2021; Colpo et al. 2022). Particularly, acclimation was obtained by maintaining plants for at least 3 weeks under contrasting light regimes in terms of photosynthetic photon fluence rate (PPFR) and light quality: high light (HL), exposure to direct sunlight, maximum PPFR > 800 μmol m^−2^ s^−1^; mid‐shade and deep‐shade, obtained with sheltering by upper plants, to reach maximum PPFR of ca. 80 or < 10 μmol m^−2^ s^−1^, respectively. Some pots were delivered to the Slovak University of Agriculture in Nitra, where they were placed in a greenhouse under similar conditions (N 48°18′15″, E 18°05′58″). For all analyses, terminal branches, that is, ca. 2 cm‐long stems were used, including the first dichotomic ramification from the vegetative apex. In particular, the subapical portion was probed.

### 2.2 Continuous Excitation Chlorophyll *a* Fluorescence

The fast chlorophyll *a* fluorescence emission was analyzed in vivo with a continuous excitation Handy‐PEA fluorimeter (Hansatech Instruments Ltd.). Terminal branches were cut and placed on wet chromatographic paper to avoid water stress during the measurements. The PSII functional antenna size was estimated by applying the protocol described by Dinç et al. (2012) and Bielczynski et al. (2016). After 30 min dark acclimation, a series of 10 saturation pulses (SP) of 0.6 s with increasing PPFR from 200 to 3500 μmol m^−2^ s^−1^ was applied, with 10 min darkness interposed between each SP. Under continuous excitation conditions, the fluorescence signal intensity is proportional to the intensity of excitation light. Therefore, the fluorescence values were normalized on the PPFR of each SP and the fluorescence values were sampled at 300 μs (*F*_300_/PPFR). *F*_300_/PPFR values were plotted against PPFR and the slope of the regression line was evaluated. The slope is proportional to the absorption cross section of PSII and, therefore, provides a precise estimate of the PSII functional antenna size in the dark‐acclimated state. In the case of saturation of or a decrease in *F*_300_/PPFR at high PPFR, the points deviating from the linearity were excluded from the linear fitting.

The PSII excitonic connectivity was analyzed in transients recorded at 1000 μmol m^−2^ s^−1^, which met the requirement of reaching the maximum variable fluorescence (Stirbet and Govindjee 2011; Laisk and Oja 2013). The occurrence of PSII excitonic connectivity was evaluated according to Strasser and Stirbet (2001). Assuming that separate (unconnected) PSII units exhibit an exponential rise in fluorescence between 20 and 300 μs, the method quantifies the deviation of the transient recorded in this interval from a hypothetical exponential rise within the same interval. The deviation defines the sigmoidal character of the curve and was quantified as the curvature constant *C* sampled between 120 and 140 μs (L band). *C* was used to calculate the overall PSII grouping probability *p*_*2G*_ (Strasser and Stirbet 2001) and the Joliot's connectivity parameter *p* (Joliot and Joliot 1964, 1972). *p*_*2G*_ quantifies the probability that an exciton moves between open PSII units, while *p* is the probability that an exciton moves from a closed PSII to another PSII in any state (Stirbet 2013).

The same transients were double normalized between the steps O (minimum fluorescence at 20 μs) and P (maximum fluorescence), and the following parameters related to the electron transport were calculated based on normalized fluorescence values *V*: Δ*V*_J_, corresponding to 1 − *V*_J_, where J is the transient inflection at 2 ms; Δ*V*_I_, corresponding to 1 − *V*_I_, where I is the transient inflection at 30 ms; *Sm*, the normalized area above the transient (Stirbet and Govindjee 2011).

### 2.3 Modulated Chlorophyll *a* Fluorescence With Simultaneous Measurement of P700 Redox State

The state of PSI and PSII photochemistry was analyzed simultaneously with a Dual PAM‐100 (Walz) equipped with a chlorophyll fluorescence unit (detection at *λ* > 700 nm) and P700 dual wavelength (Δ*I*_830–875nm_/*I*_830–875nm_) unit, as described by Klughammer and Schreiber (1994).

The plants were acclimated for 30 min in a dark box and then for 2 min in the measuring head. After the dark‐acclimation, an SP (width 700 ms, intensity 3000 μmol m^−2^ s^−1^) was applied for the analysis of chlorophyll *a* fluorescence emission and P700 absorptivity. The values in dark‐acclimated state were measured and used for reference for the subsequent light treatments: the minimum fluorescence *F*_*0*_; the maximum fluorescence *F*_*M*_ induced by the SP; the maximum P700 absorption signal determined by applying a SP after 10 s of far‐red pre‐illumination (720 nm) and corresponding to the difference between the maximum and minimum P700^+^ signal ΔP_M_, hereafter simply indicated as P_M_. The fast kinetics of chlorophyll fluorescence induction and P700 oxidation were recorded with a sampling rate of 3.33 ms^−1^ (one value every 0.3 ms during the 700 ms‐long SP).

After recoding the dark‐acclimated parameters, for the full activation of the photosynthetic process, the branch was exposed to a light intensity of 217 μmol m^−2^ s^−1^ for 10 min (red actinic light, 635 nm); the same condition is permissive to the stomatal opening (Ferroni, Brestič, et al. 2021). During the light induction, an SP was applied every 30 s. Once the light‐acclimated steady state was reached, the sample was acclimated to a low light condition: 7 μmol m^−2^ s^−1^ for 5 min, 14 μmol m^−2^ s^−1^ for 4 min, 23 μmol m^−2^ s^−1^ for 3 min. Subsequently, the sample was exposed to increasing irradiance from 38 to 431 μmol m^−2^ s^−1^ through steps lasting for 3 min, and from 532 to 1089 μmol m^−2^ s^−1^ through steps lasting for 2 min (Figure S1). After each light step, beside the light‐acclimated fluorescence *F* and the P700^+^ absorbance signal P, an SP followed by far‐red exposure (8 s) allowed the determination of *F*_*M*_′, *F*_*0*_′ and P_M_′.

### 2.4 Calculation of Quantum Yields and Electron Flows

In the dark‐acclimated state, the *F*_*V*_/*F*_*M*_ ratio was calculated, where *F*_*V*_ = *F*_*M*_ *−F*_*0*_. *F*_*V*_/*F*_*M*_ was assumed as a proxy for the maximum quantum yield of PSII photochemistry. The modulated chlorophyll fluorescence values were used to calculate the complementary quantum yields in the light‐acclimated state as in Hendrickson et al. (2004): Y(PSII) = (*F*_*M*_−*F*)/*F*_*M*_′, the actual PSII quantum yield; Y(NO) = *F*/*F*_*M*_, the quantum yield of non‐regulatory energy dissipation in PSII; Y(NPQ) = 1−Y(PSII)‐Y(NO), the quantum yield of regulatory thermal dissipation. Stern–Volmer‐type NPQ equals the Y(NPQ)/Y(NO) ratio (Lazár 2015).

P, P_M_, P_M_′ levels of P700 oxidation state were combined to calculate the complementary quantum yield of PSI as in Klughammer and Schreiber (1994): Y(PSI) = (P_M_′ − P)/P_M_, the effective quantum yield (efficiency) of PSI photochemistry; Y(ND) = P/P_M_, the quantum yield of the non‐photochemical dissipation in donor‐side limited PSI, or the fraction of P700 that is oxidized at given state, P700^+^/P700 total; Y(NA) = (P_M_ − P_M_′)/P_M_, the quantum yield of the non‐photochemical dissipation in acceptor‐side limited PSI, that is, an estimate of PSI overreduction due to a lack of downstream oxidized electron acceptors.

Preliminarily to the calculation of electron transport rates, the excitation distribution between the two photosystems was calculated according to Huang et al. (2012). Under low light conditions, plants have a minimal need to exploit the CEF (Miyake et al. 2005; Joliot and Joliot 2006; Huang et al. 2011; Zivcak et al. 2013). We assumed that at 23 μmol m^−2^ s^−1^, CEF was negligibly induced, that is, the ETR(II) and ETR(I) had approximately the same magnitude, and the energy distribution to PSII d_II_ was calculated (Sukhov et al. 2015):

$$d_{\mathrm{II}}=\frac{1}{\frac{Y\left(\text{PSII}\right)}{Y\left(\mathrm{PSI}\right)}+1}$$

The electron transport through each photosystem was calculated as:

$$\mathrm{ETR}\left(\mathrm{II}\right)=\text{PPFR}\cdot Y\left(\text{PSII}\right)\cdot d_{\mathrm{II}}\cdot 0.84$$

$$\mathrm{ETR}\left(I\right)=\text{PPFR}\cdot Y\left(\mathrm{PSI}\right)\cdot \left(1-d_{\mathrm{II}}\right)\cdot 0.84$$

The ETR‐irradiance curves were treated analytically using Platt's exponential equation with photoinhibition (Platt et al. 1990). The CEF was quantified as the excess of electron flow through PSI as compared to the electron inflow into the chain from PSII and was expressed by the difference $\mathrm{CEF}=\mathrm{ETR}\left(I\right)-\mathrm{ETR}\left(\mathrm{II}\right)$. The CEF‐irradiance curves were fitted with Hill's function. In all cases, the curves are shown within the irradiance range of 23–661 μmol m^−2^ s^−1^ because for higher irradiance, the changes in fluorescence and P700 absorbance values became too low for a reliable analytical treatment.

### 2.5 Calculation of Q_A_ Redox State

The fraction of reduced Q_A_ (Q_A_^−^/Q_A_total) was calculated from chlorophyll fluorescence parameters during light curves according to a “puddle model” and a “connected units model” of PSII organization. For the former, it was evaluated as 1 − *qP*, where *qP* is the photochemical fluorescence quenching (Schreiber and Bilger 1987; Weis and Berry 1987; Genty et al. 1989):

$$\mathrm{qP}=\frac{F_{M}^{\prime }-F}{F_{M}^{\prime }-{F_{O}}^{\prime }}$$

In a connected PSII units model, Q_A_^−^/Q_A_total was evaluated as 1 − *qCU*, defining *qCU* as follows (Kramer et al. 2004):

$$\mathrm{qCU}=\frac{F_{M}^{\prime }-F}{{\left(\frac{p}{1-p}\right)\left(F-{F_{O}}^{\prime }\right)+F}_{M}^{\prime }-{F_{O}}^{\prime }}$$

where *p* is the Joliot's connectivity parameter derived from the OJIP transients (see Section 2.2).

### 2.6 Measurements of Electrochromic Bandshift

A LED‐based JTS 10 spectrometer (BioLogic) was used to measure the absorbance changes denoted as the electrochromic bandshift (ECS) according to Joliot and Joliot (2002) and adapting the protocol described by Zivcak, Kalaji, et al. (2014). An uncut terminal branch was inserted into a handmade plastic sample holder, which was positioned in the spectrometer. Before ECS measurements, a rapid rise in 520 nm absorbance (ECS_ST_) was induced in the leaf by a saturating single‐turnover flash. Subsequently, an orange light (630 nm) of 150 μmol m^−2^ s^−1^ was driven to the sample for 10 min to reach steady state before the measurements of ECS decay. Subsequently, the actinic light was switched off (dark pulse) and the ECS decay was measured by monitoring the absorption changes at 520 nm for 30 s. The light was immediately switched on again for 3 min and the ECS decay at the same sample was then measured at 546 nm. The signal measured at 546 nm was subtracted from that measured at 520 nm to deconvolute the ECS signal from the absorption changes associated with redox changes related to the electron transport activity of the cyt*b*_*6*_ *f* complex (Joliot and Joliot 2002). The ECS signal at 520 nm was normalized against the ECS_ST_ to account for even minor differences in light absorption between samples (Takizawa et al. 2007). The rapid decay (within 150 ms) of the normalized ECS signal during the dark pulse was fit with an exponential decay, whose amplitude is an estimate of the proton‐motive force (*pmf*). The time constant of the relaxation (*τ*_*ECS*_) is inversely proportional to the proton conductivity $\left(g_{H^{+}}=\tau_{\mathrm{ECS}}^{-1}\right)$ and quantifies the ATP synthase activity of the thylakoid membrane (Sacksteder and Kramer 2000). The relative light‐driven proton flux was calculated as $\nu_{H^{+}}=\mathrm{pmf}\cdot g_{H^{+}}$ (Huang et al. 2018).

### 2.7 Transmission Electron Microscopy

Plants were transferred from the greenhouse to the laboratory and acclimated for 1 h to a PPFR of ca. 200 μmol m^−2^ s^−1^ (cool white LEDs). Subsequently, small subapical portions of branches were cut and transferred into a 10‐mL syringe, and 3 mL of 3% glutaraldehyde in 0.1 M K‐Na phosphate buffer, pH 7.2, were added. To facilitate the penetration of the fixing solution into the leaves, a slight vacuum was obtained by using the syringe plunger. After 4 h fixation at room temperature and subsequent washing with phosphate buffer, the samples were post‐fixed with 1% OsO4 in the same buffer overnight at 4°C. Dehydrated with a graded acetone series, the samples were infiltrated with Durcupan ACM epoxy resin (Colpo et al. 2023). The ultrathin sections, contrasted with lead citrate and UAR‐EMS uranyl acetate replacement stain (Electron Microscopy Sciences), were observed at the Electron Microscopy Centre, University of Ferrara, with a Talos L120C‐G2 electron microscope (ThermoFisher Scientific), operating at 120 kV under transmission mode and equipped with a 16‐megapixel Ceta camera (ThermoFisher Scientific). Periodicities in the regions of thylakoid appression (stacking repeat distance and lumen width) were determined according to Ünnep et al. (2014) by applying fast Fourier transformation (FFT) function of Fiji software on the selected area of electron micrographs taken at 17.500×.

### 2.8 Data Treatment

Statistical analyses, curve fitting, and graphical representations were performed using Origin software version 2025b (OriginLab Corporation). Comparison of L, M, and H datasets was carried out by one‐way ANOVA followed by post hoc Tukey test, in all cases fixing the threshold for statistical significance at *p* = 0.05.

## 3 Results

### 3.1 Functional Organization of PSII and Electron Carriers

**FIGURE 1:**
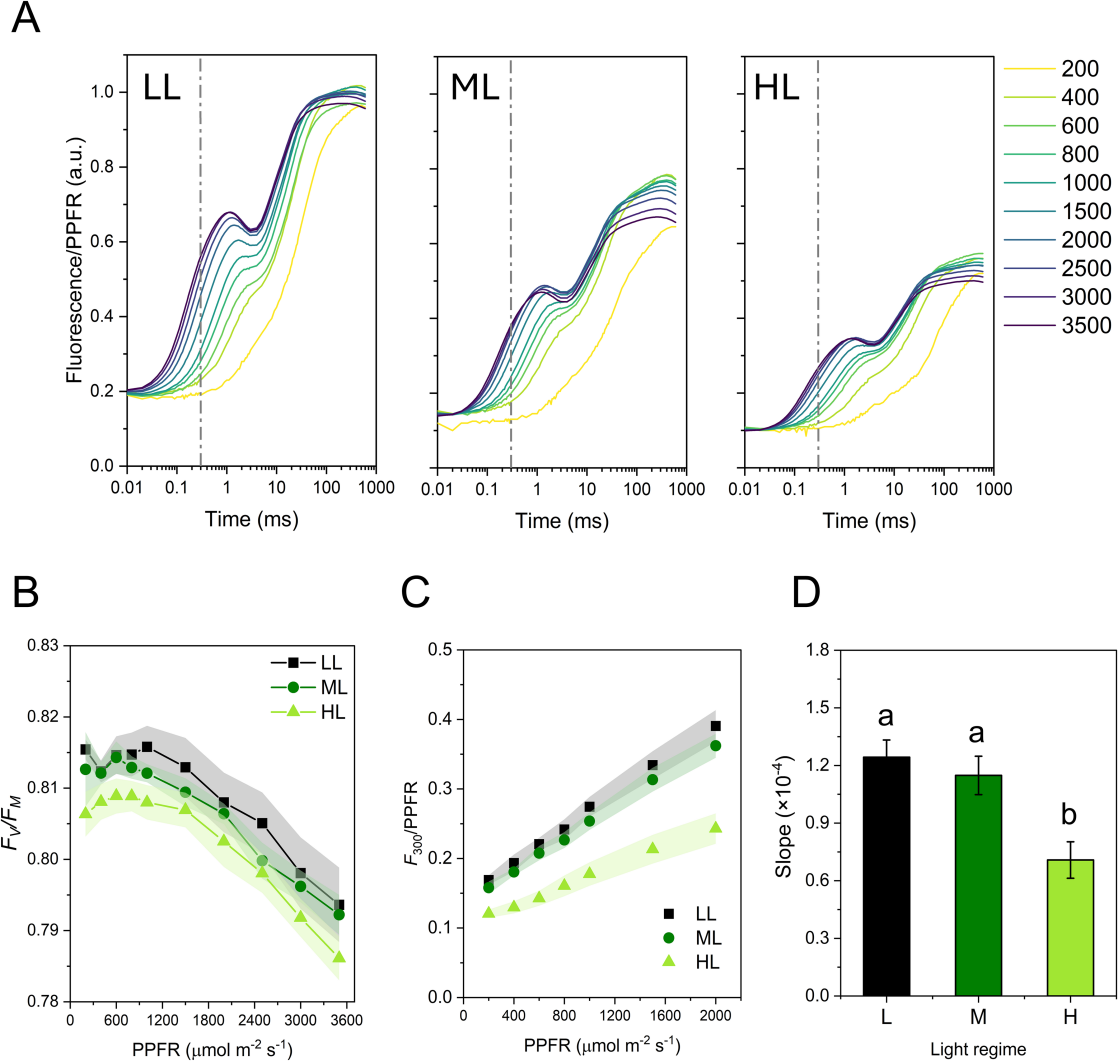
PSII antenna size determination by fast chlorophyll *a* fluorescence induction in *Selaginella martensii* plants long‐term acclimated to deep shade (LL), mid‐shade (ML), or high‐light (HL) natural regimes. (A) Representative OJIP transients recorded with excitation of increasing intensity (PPFR), as reported in the legend on the right. (B) Changes in *F*_*V*_/*F*_*M*_ values during the measuring routine shown in (A). (C) Dependence on PPFR of the fluorescence recorded at 300 μs normalized on PPFR. (D) Relative PSII antenna size evaluated as the slope of regression lines calculated from (C). In (B–C), average values are shown with SE, represented as shadowed bands, from 5 to 8 independent plants. Data in (D) are means with SE; they were treated with ANOVA and different letters indicate a significant difference with *p* < 0.05 as resulting from post hoc Tukey's test.

The PSII antenna size was determined based on the fast chlorophyll *a* fluorescence transients induced by increasing intensity of a light pulse (Figure 1A). After the origin at 20 μs, the transients presented the J step at 2–3 ms and the subsequent I inflection at ca. 30 ms. Particularly evident in LL plants was the presence of a dip when the light pulse was higher than 1000 μmol m^−2^ s^−1^. The use of repeated supersaturating pulses exposed PSII to a slight photoinhibition, as was visible in the *F*_*V*_/*F*_*M*_ decline (Figure 1B). The method by Dinç et al. (2012) was accordingly applied to transients recorded with excitation up to 2000 μmol m^−2^ s^−1^ (Figure 1C). PSII antenna size was the same in LL and ML plants, while it was reduced by ca. 40% in HL plants (Figure 1D). With respect to PSII connectivity, the sigmoidal character of the transients recorded at 1000 μmol m^−2^ s^−1^ was similar in all plants, and the excitonic connectivity parameters *p*_*2G*_ and *p* did not differ significantly (Table 1 and Figure S2).

**TABLE 1:** Parameters derived from fast chlorophyll *a* fluorescence kinetics measurement in *Selaginella martensii* plants long‐term acclimated to deep shade (LL), mid‐shade (ML), or high‐light (HL) natural regimes.

Parameter	LL	ML	HL
*F*_*V*_/*F*_*M*_	0.816 ± 0.003^a^	0.812 ± 0.001^a^	0.805 ± 0.003^a^
*F*_*V*_/*F*_0_	4.43 ± 0.09^a^	4.32 ± 0.04^a^	4.14 ± 0.09^a^
*C*	0.67 ± 0.03^a^	0.81 ± 0.08^a^	0.67 ± 0.03^a^
*p*_*2G*_	0.15 ± 0.005^a^	0.19 ± 0.02^a^	0.16 ± 0.01^a^
*p*	0.40 ± 0.01^a^	0.44 ± 0.02^a^	0.40 ± 0.01^a^
Δ*V*_J_	0.535 ± 0.009^a^	0.533 ± 0.007^a^	0.530 ± 0.019^a^
Δ*V*_I_	0.140 ± 0.008^b^	0.191 ± 0.013^ab^	0.200 ± 0.016^a^
Δ*V*_I_/Δ*V*_J_	0.261 ± 0.016^b^	0.358 ± 0.025^a^	0.378 ± 0.020^a^
*Sm*	16.6 ± 0.7^b^	22.9 ± 1.3^a^	23.5 ± 1.7^a^

*Note:* Values (means with SE) were obtained upon induction with a 0.6 s saturation pulse of 1000 μmol m^−2^ s^−1^ from 5 (LL, ML) to 8 (HL) independent plants. Parameters: *F*_*V*_/*F*_*M*_, maximum quantum yield of PSII; *F*_*V*_/*F*_*0*_, index of maximum photochemical capacity of PSII; *C*, curvature constant of initial phase of the O–J curve; *p*_*2G*_, overall PSII grouping probability; *p*, Joliot's PSII connectivity parameter; Δ*V*_J_, complement of the O–P double‐normalized fluorescence at 2 ms; Δ*V*_I_, complement of O–P double‐normalized fluorescence at 30 ms; *Sm*, normalized area above the transient. Data were treated with ANOVA and different letters indicate a significant difference with *p* < 0.05 as resulting from post hoc Tukey's test.

From the same transients, information was obtained on the pools of electron transporters. Δ*V*_J_ was identical in the three plant groups, indicating the same size of the PQ pool (Tóth et al. 2007). Differently, Δ*V*_I_ and, accordingly, also Δ*V*_I_/Δ*V*_J_ increased by ca. 40% from LL to ML and HL, corresponding to an enlargement of the electron acceptor pool size of PSI (Zivcak et al. 2015; Ferroni et al. 2022). Th *Sm* parameter, proportional to the number of electron carriers per electron transport chain, changed accordingly (Stirbet and Govindjee 2011). Because Δ*V*_I_ can also reflect the relative content of PSI (Ceppi et al. 2012; Pollastrini et al. 2020), we checked the P_M_ values obtained with Dual‐PAM. PSI content, normalized for leaf chlorophyll, was approximately twice as high in ML plants and three times higher in HL plants compared to LL plants (Table S1), consistent with previous findings (Ferroni et al. 2016).

**FIGURE 2:**
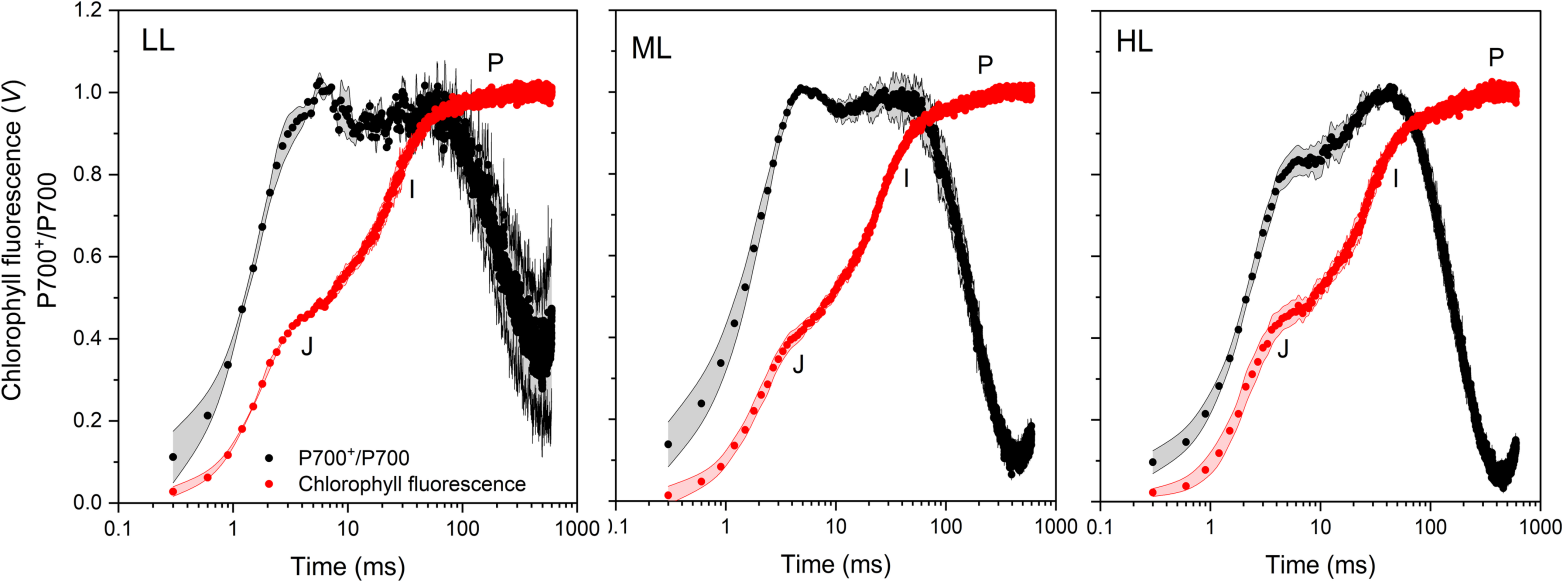
Simultaneous kinetics of fast chlorophyll *a* fluorescence and relative amount of P700^+^ in *Selaginella martensii* plants long‐term acclimated to deep shade (LL), mid‐shade (ML), or high‐light (HL) natural regimes. Normalized chlorophyll *a* fluorescence and P700^+^ accumulation kinetics are shown on a logarithmic time scale. The noticeable steps of the fluorescence transient are indicated. Average values are shown with SE, represented as shadowed bands, from 3 to 6 independent plants.

To obtain further information on the electron carriers, we compared the changes in P700 oxidation kinetics during the fast chlorophyll fluorescence rise induced by a SP (Figure 2). Normally, accumulation of P700^+^ occurs progressively up to a transitory steady state approaching the I step of the fluorescent transient; subsequently, the electrons made available by reduced PQ cause the re‐reduction of P700^+^ (e.g., Zivcak, Kalaji, et al. 2014). However, in LL and ML plants, the relative P700^+^ amount saturated soon, already at the level of the J step. In HL plants, it reached ca. 80% at the J step, and the accumulation rate was 35% slower than in LL samples, indicating a smaller PSI antenna size (Figure 2 and Table S1). The P700^+^ accumulation conveyed the idea that the majority of photon energy excited PSI in all plant groups. During the I‐P phase, the re‐reduction of P700^+^ was typically slow in *S. martensii* (Colpo et al. 2023); however, the rate of P700^+^ re‐reduction increased conspicuously from LL to HL plants, reflecting a higher availability of electron carriers in the latter (Figure 2 and Table S1). The P700 kinetics during a SP can also bring information on the consumption of electrons downstream of PSI by FLVs (Ilík et al. 2017). Especially if represented on a linear time scale, P700 kinetics showed a relatively slow re‐oxidation from ca. 400 ms, which was barely visible in LL plants, but more evident in ML plants and especially in HL plants, suggesting the accumulation of FLVs (Figure S3).

### 3.2 Thylakoid Functioning and Ultrastructure in the Light‐Acclimated State

**FIGURE 3:**
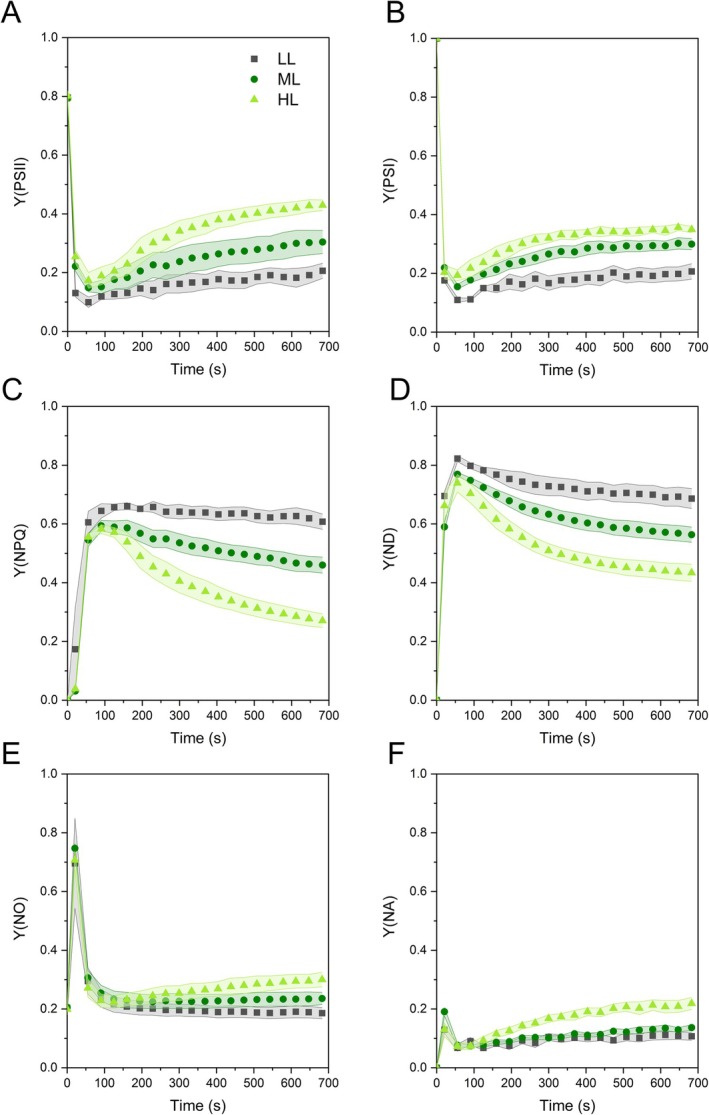
Slow kinetics of chlorophyll *a* fluorescence parameters during light induction (217 μmol m^−2^ s^−1^) in *Selaginella martensii* plants long‐term acclimated to deep shade (LL), mid‐shade (ML), or high‐light (HL) natural regimes. (A) Quantum yield of PSII photochemistry, Y(PSII). (B) Quantum yield of PSI photochemistry, Y(PSI). (C) Non‐photochemical quantum yield of the regulatory thermal dissipation in PSII, Y(NPQ). (D) Non‐photochemical quantum yield of PSI limited at the donor side, Y(ND). (E) Non‐photochemical quantum yield of the non‐regulatory thermal dissipation in PSII, Y(NO). (F) Non‐photochemical quantum yield of PSI limited at the acceptor side, Y(NA). Average values are shown with SE, represented as shadowed bands, from 4 to 5 independent plants.

To test the functioning of the thylakoid membrane in the light, we induced photosynthesis with ca. 200 μmol m^−2^ s^−1^ actinic light. During 10 min induction, Y(PSII) and Y(PSI) changed almost in parallel, reaching a quasi‐steady state; HL plants had twice the capacity to maintain photochemical photosystem activity than LL plants (Figure 3A,B). With respect to the protective non‐photochemical processes as indicated by Y(NPQ) and Y(ND), after a similar level of induction during the first minute, the samples stabilized with a gradient opposite to that of Y(PSII) and Y(PSI), respectively (Figure 3C,D). Low Y(NO) and Y(NA) indicated that electron flow was well controlled in all plants (Figure 3E,F).

**FIGURE 4:**
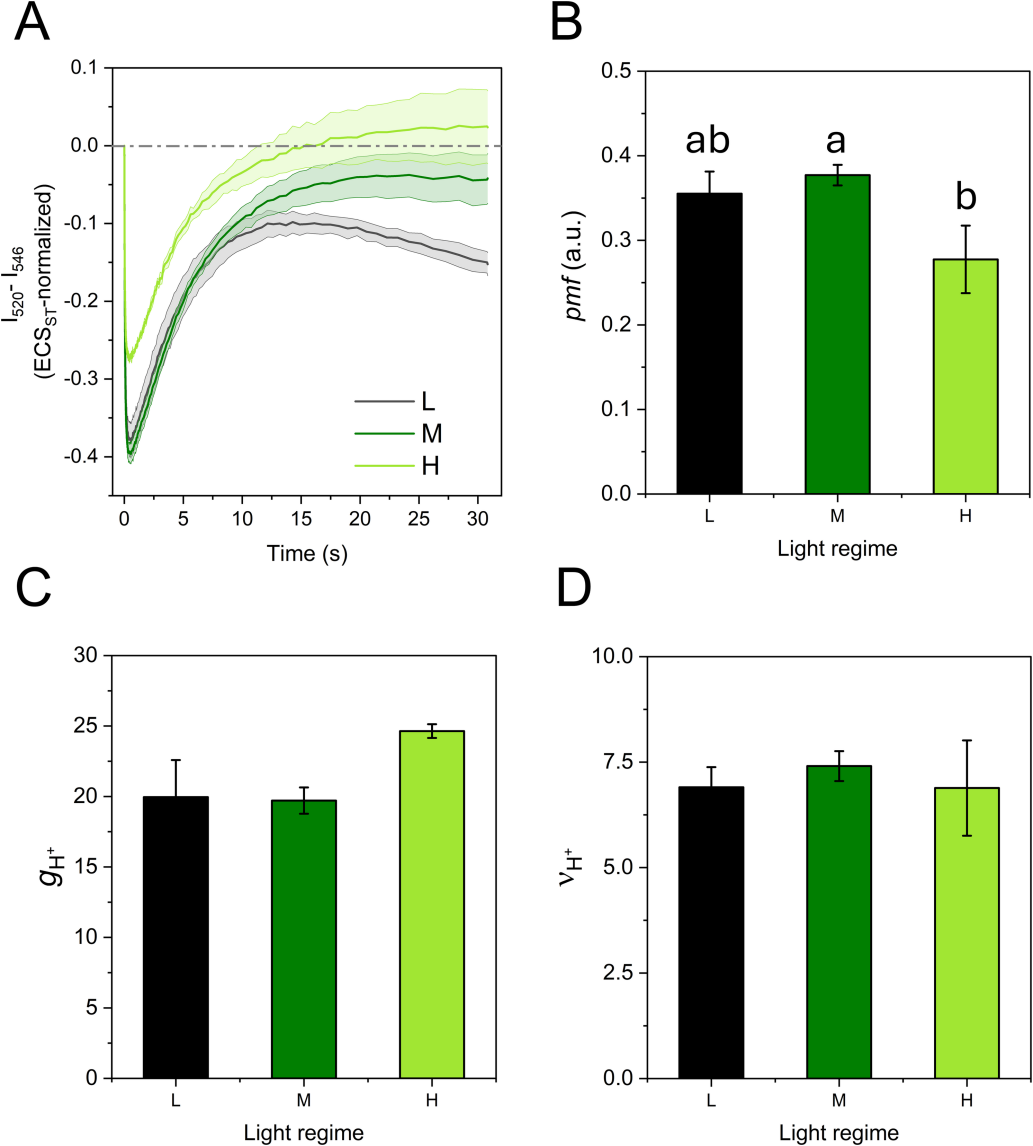
Electrochromic bandshift (ECS) analysis in *Selaginella martensii* plants long‐term acclimated to deep shade (LL), mid‐shade (ML), or high‐light (HL) natural regimes. (A) Dark‐interval relaxation kinetics of ECS after 10 min illumination of leaves by 150 μmol photons m^−2^ s^−1^. (B) Proton‐motive force (*pmf*) evaluated as the amplitude of ECS signal. (C) Proton conductance *g*_*H+*_, an estimate of ATP synthase activity. (D) Total proton flux through the photosynthetic membrane, *ν*_H+_. Average values are shown with SE, represented as shadowed bands (A) or error bars (B–D), from 4 to 7 independent plants. Data were treated with ANOVA and different letters indicate a significant difference with *p* < 0.05 as resulting from post hoc Tukey's test.

Using the ECS method under similar conditions, the *pmf* was found to be similar in ML and LL plants, but ca. 20% lower in HL plants, therefore in line with smaller Y(NPQ) (Figure 4A,B); lower *pmf* in HL plants was associated with higher proton conductivity *g*_H+_, that is, higher ATP synthase activity (ML‐HL Student's *t*‐test, *p* < 0.01; Figure 4C). The total proton flux *ν*_H+_ was similar in the three plant groups (Figure 4D). Following the rapid decay of the ECS signal, HL and ML plants exhibited a slower rise that returned to the dark baseline, usually attributed to *pmf* driven solely by ΔpH. In LL plants, the inversion was incomplete, which could indicate a non‐negligible electric field component ΔΨ of the *pmf* (Cruz et al. 2005).

**FIGURE 5:**
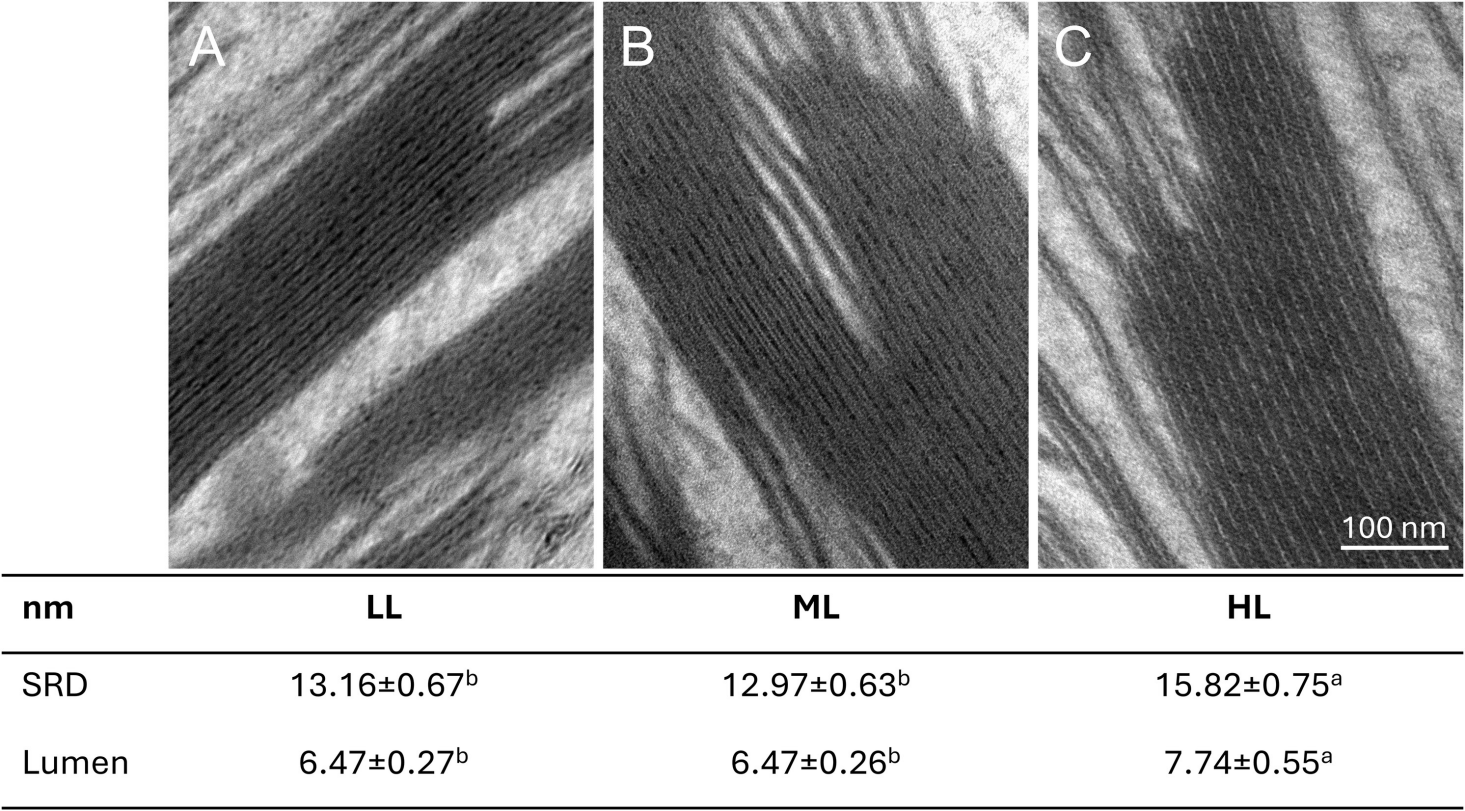
Thylakoid ultrastructure in *Selaginella martensii* plants long‐term acclimated to deep shade (LL), mid‐shade (ML), or high‐light (HL) natural regimes. Plants were exposed to 200 μmol photons m^−2^ s^−1^ for 1 h. (A–C) Representative views of the thylakoids in LL (A), ML (B) and HL (C) plants; note the dark and narrow lumen in LL and ML samples, while it is lighter in HL sample. Underneath, the table reports the corresponding determinations of the stacking repeat distance (SRD) and lumen width. Average values are shown with SD, from *N* = 47–63 grana. Data were treated with ANOVA and different letters indicate a significant difference with *p* < 0.05 as resulting from post hoc Tukey's test.

Most photosynthetic activity in *S. martensii* leaves is due to the giant chloroplast, hosted in each upper epidermal cell of the microphyll (Colpo et al. 2023). The organelle was a bizonoplast in ML and HL conditions, with a lamellar region at the top and a prominent granal region below (for the definition of a bizonoplast, see Sheue et al. 2007). In LL plants, the organization of the thylakoids was pseudo‐lamellar overall without clear zonation (Figure S5; Ferroni et al. 2016). The thylakoid *SRD* was minute and the same in ML and LL plants (13 nm), while it increased visibly in HL plants (16 nm; Figure 5). The increase in *SRD* was partly due to an expanded thylakoid lumen. In LL and ML plants, the lumen was very thin (6.5 nm) and electron‐dense, while it was 1.3 nm larger and clear in HL thylakoids (Figure 5A–C).

### 3.3 Photochemical Activity of PSII and PSI Under Increasing Irradiance

After reaching a steady state of photosynthesis in the light‐acclimated state (light induction, see 3.2), the plants were acclimated to low irradiance and then probed for PSII and PSI activity through increasing light steps. Examples of rough chlorophyll fluorescence and P700^+^ traces are shown for representative LL, ML, and HL plants in Figure S4. Particularly, the hypothesis was tested whether in HL plants, higher amounts of PSI and electron carriers (Table 1 and Table S1) led to higher levels of CEF than in LL plants.

**FIGURE 6:**
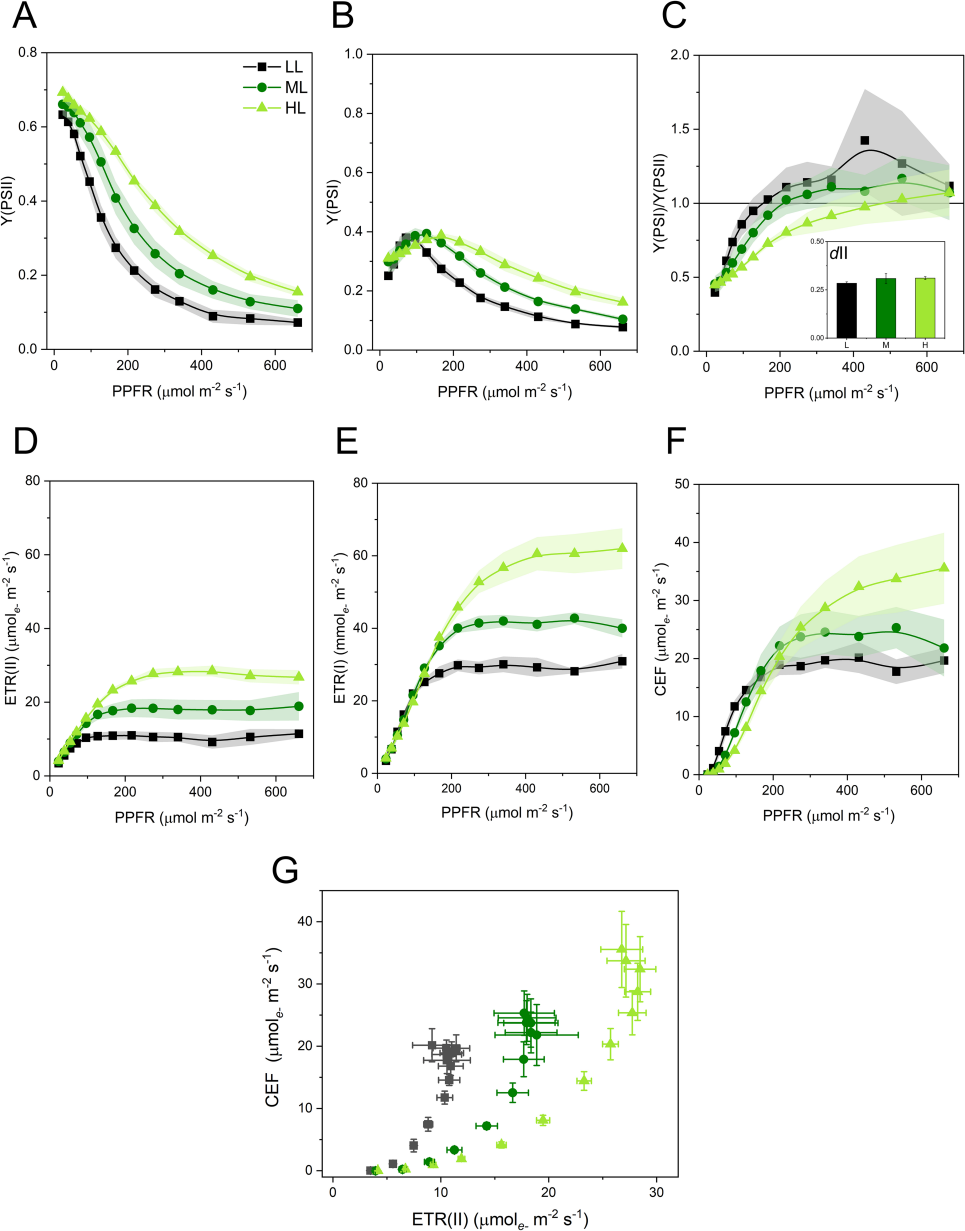
Light curves of photosystem activity in *Selaginella martensii* plants long‐term acclimated to deep shade (LL), mid‐shade (ML), or high‐light (HL) natural regimes. (A) Quantum yield of PSII photochemistry, Y(PSII). (B) Quantum yield of PSII photochemistry, Y(PSI). (C) Y(PSII)/Y(PSII) ratio; in the insert, energy distribution to PSII, *d*II. (D) Linear electron transport rate, that is, electron transport through PSII, ETR(II). (E) Electron transport through PSI, ERT(I). (F) Cyclic electron flow, CEF. (G) Co‐variation of ETR(II) and CEF. Average values are shown with SE, represented as shadowed bands, from 4 to 5 independent plants.

Y(PSII) presented a typical light‐intensity dependence, decreasing progressively from low to high light according to a well visible HL‐ML‐LL gradient (Figure 6A). Y(PSI) increased from low to medium irradiance and then progressively decreased following the same gradient as Y(PSII) (Figure 6B). The co‐variation of Y(PSI) and Y(PSII) has been commonly used to assess the occurrence of CEF (e.g., Livingston et al. 2010; Zivcak et al. 2013, 2019; Zivcak, Brestic, et al. 2014; Zivcak, Kalaji, et al. 2014; Kono et al. 2014; Yamori et al. 2016; Nakano et al. 2019). Particularly, CEF is revealed by Y(PSI) > Y(PSII), and changes in Y(PSI)/Y(PSII) ratio are interpreted as variations in the relative importance of CEF. However, recent reports have challenged the concept that Y(PSI) > Y(PSII) may depend on CEF. One reason can be the need for P_M_ correction because of sustained PSI photoinhibition, as occurs in boreal evergreen conifers during the winter (Grebe et al. 2024). Another reason seems inherent to the SP methodology; in particular, Furutani et al. (2022) showed that enhanced states of PSI donor‐side limitation can lead to overestimation of Y(PSI). With either failed P_M_ determination or high Y(ND), the CEF inferred from Y(PSI) > Y(PSII), or Y(PSI)/Y(PSII) > 1, would be artefactual. However, in *S. martensii* , the Y(PSI)/Y(PSII) ratio was consistently as low as ~0.4 at low light, irrespective of the growth light regime, and gradually increased at more intense irradiance until stabilizing at ~1 (Figure 6C). This result excludes biased P_M_ determinations and is also hardly reconciliable with methodological pitfalls leading to Y(PSI) overestimation. Therefore, we have chosen a conservative approach and have conformed to the mainstream interpretation of the co‐variation of Y(PSI) and Y(PSII). In relative terms, the increasing Y(PSI)/Y(PSII) suggested a more active CEF; nonetheless, the common assumption of equal excitation distribution between PSI and PSII would have led to a negative difference between ETR(I) and ETR(II).

There is a large consensus that at nearly limiting light intensity plants induce minimal CEF, so that ETR(I) approximately equals ETR(II) (Laisk et al. 2005, 2010; Miyake et al. 2005; Joliot and Joliot 2006; Huang et al. 2011, 2012; Zivcak et al. 2013; Sukhov et al. 2015). In such a case, different photochemical rates of PSI and PSII are explained by unequal excitation distribution between the two photosystems. Under low light, we estimated that ~70% of the excitation energy collected by the antenna system was driven to PSI, irrespective of the growth light regime (Figure 6C insert). Excitation partitioning between PSI and PSII was then used to calculate ETR(I) and ETR(II). A distinct gradient in ETR(I) and ETR(II) was evident according to the growth light regime (Figure 6D,E). Compared to LL, HL plants gained a 2.5‐ and 2‐times capacity of electron transport through PSII and PSI, respectively, and the saturating irradiance *E*kII of ETR(II) doubled from LL to HL plants. The apparent quantum efficiencies αI and αII were unaffected by the growth light regime, but αI consistently exceeded αII, on average 0.30 versus 0.22 mol electrons mol^−1^ photons, respectively (Table 2).

**TABLE 2:** Photosynthetic electron transport in *Selaginella martensii* plants long‐term acclimated to deep shade (LL), mid‐shade (ML), or high‐light (HL) natural regimes.

Parameter	LL	ML	HL
ETR(II) max (μmol *e* ^*−*^ m^−2^ s^−1^)	11.6 ± 0.9^c^	18.9 ± 2.1^b^	28.5 ± 0.7^a^
αII (mol *e* ^*−*^ mol^−1^ photons)	0.210 ± 0.012^a^	0.239 ± 0.022^a^	0.233 ± 0.011^a^
*E*κII (μmol photons m^−2^ s^−1^)	55.9 ± 5.6^b^	81.2 ± 11.8 ^b^	123.0 ± 6.7 ^a^
ETR(I) max (μmol *e* ^*−*^ m^−2^ s^−1^)	31.5 ± 2.0^b^	43.8 ± 3.3^b^	63.3 ± 5.6^a^
αI (mol *e* ^*−*^ mol^−1^ photons)	0.297 ± 0.006^a^	0.315 ± 0.007^a^	0.297 ± 0.008^a^
CEF max (μmol *e* ^*−*^ m^−2^ s^−1^)	19.9 ± 1.7^a^	24.9 ± 3.9^a^	34.8 ± 5.9^a^
*n*_H_	3.12 ± 0.32^a^	3.67 ± 0.28^a^	2.75 ± 0.10^a^
CEF max/ETR(II) max	1.74 ± 0.19^a^	1.45 ± 0.35^a^	1.23 ± 0.23^a^

*Note:* Values (means with SE) were obtained from simultaneous chlorophyll *a* fluorescence and P700 analyses during light curves (*N* = 4–5 independent plants). Parameters: ETR(II) max, maximum electron transport rate through PSII or linear electron transport; αII, quantum efficiency of electron transport through PSII; *E*kII, minimum saturating irradiance of ETR(II); ETR(I) max, maximum electron transport rate through PSI; αI, quantum efficiency of electron transport through PSI; CEF max, maximum cyclic electron transport, as the difference between ETR(I) max and ETR(II) max; *n*_H_ is Hill's coefficient obtained from the CEF‐irradiance curves. Data were treated with ANOVA and different letters indicate a significant difference with *p* < 0.05 as resulting from post hoc Tukey's test.

Because in all samples there was a strong prevalence of ETR(I) over ETR(II), the ETR(I)‐ETR(II) difference was calculated as a proxy for CEF. The CEF‐irradiance relationship was sigmoidal in all plant groups, with a similar degree of sigmoidicity, determined as Hill's coefficient *n*_H_ (Figure 6F and Table 2). However, induction of CEF occurred intensely already at low light in LL but was soon saturated. Conversely, in HL plants, the intense CEF induction occurred at higher irradiance and seemed not to saturate. In HL plants, the comparative analysis of LEF, as ETR(II), and CEF showed a quasi‐linear increase of CEF with LEF up to ca. 10 μmol electron m^−2^ s^−1^, followed by a much more intense activation of CEF (Figure 6G). The quasi‐linear phase was less evident in ML plants and negligible in LL plants. Although ANOVA showed no significant difference in maximum CEF (Table 2), a Student's *t*‐test between LL and HL plants was close to significant (*p* = 0.052) with a notable biological difference in their CEF induction capacity. The maximum CEF increased indeed by 25% and 75% in ML and HL plants, respectively, as compared to LL plants. In all plant groups, maximum CEF overcame LEF, that is, CEF/ETR(II) > 1, which was particularly evident for the LL plants (Figure 6F,G and Table 2).

### 3.4 CEF and Control of Photosynthetic Electron Poise

The effectiveness of photosynthetic electron poise control was assessed by comparatively evaluating the non‐photochemical quantum yields complementary to Y(PSI) and Y(PSII). Light curves of Y(NPQ), Y(NO), Y(ND) and Y(NA) are reported in Figure S6. Y(NPO), Y(ND) and Y(NA) showed expected gradients from LL to HL plants; particularly the former promptly induced higher levels of photoprotective Y(NPQ) and Y(ND) at low irradiance, while the latter kept higher levels of acceptor‐side limitation of PSI. Nevertheless, at high irradiance, Y(NPO) and Y(ND) were converging to high levels in all plants. Y(NO) was monotonically decreasing in LL plants, but not in ML and even less in HL plants, and all approached similar steady‐state values at high irradiance.

**FIGURE 7:**
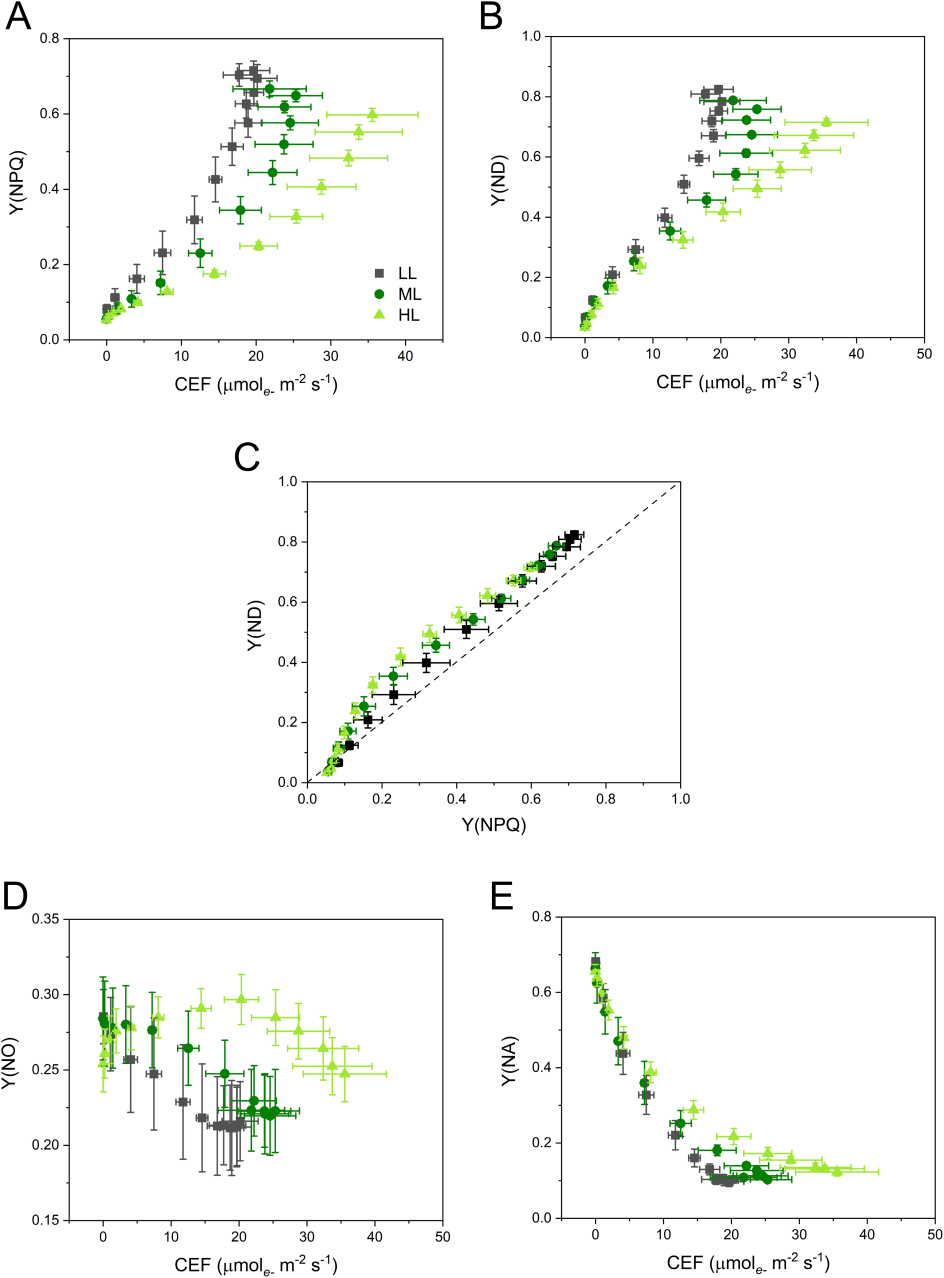
Relationship between non‐photochemical processes and cyclic electron flow (CEF) in *Selaginella martensii* plants long‐term acclimated to deep shade (LL), mid‐shade (ML), or high‐light (HL) natural regimes. The parameters are quantum yields as in Figure 3. Average values are shown with SE from 4 to 5 independent plants.

Considering that CEF is known to promote the thermal dissipation of excess energy and the donor‐side limitation of PSI (Munekage et al. 2002, 2004; Yamamoto and Shikanai 2018), the corresponding covariations of Y(NPQ) and Y(ND) with CEF were examined (Figure 7A,B). With respect to Y(NPQ), the three plant groups diverged already upon the initial induction of CEF (Figure 7A). Differently, CEF rates up to 10 μmol electrons m^−2^ s^−1^ were accompanied by an identical linear increase in Y(ND) in all plants. The subsequent Y(ND) divergence between plant groups was similar to the Y(NPQ) trends and led to higher PSI donor limitation in LL than in HL plants (Figure 7B). The covariations with CEF evidenced that Y(NPQ) and Y(ND) trends, though very similar, were not identical. This aspect was checked by plotting Y(ND) against Y(NPQ) and, in all cases, the level of P700 oxidation tended to be slightly higher than the induction of Y(NPQ) (Figure 7C). Therefore, not only was the injection of electrons into the chain restricted by NPQ, but other mechanisms were involved in the prominent accumulation of P700^+^. The Y(NO)‐CEF relationship was negative in LL and ML plants, but not in HL plants, in which CEF rates up to 20 μmol electrons m^−2^ s^−1^ were instead associated with increased Y(NO) (Figure 7D). The effectiveness of electron removal downstream of PSI, indicated by Y(NA), was similar in all plants for low values of CEF, but different at high CEF values, with a tendency to PSI overreduction in HL plants despite their capacity to develop higher CEF rates (Figure 7E).

**FIGURE 8:**
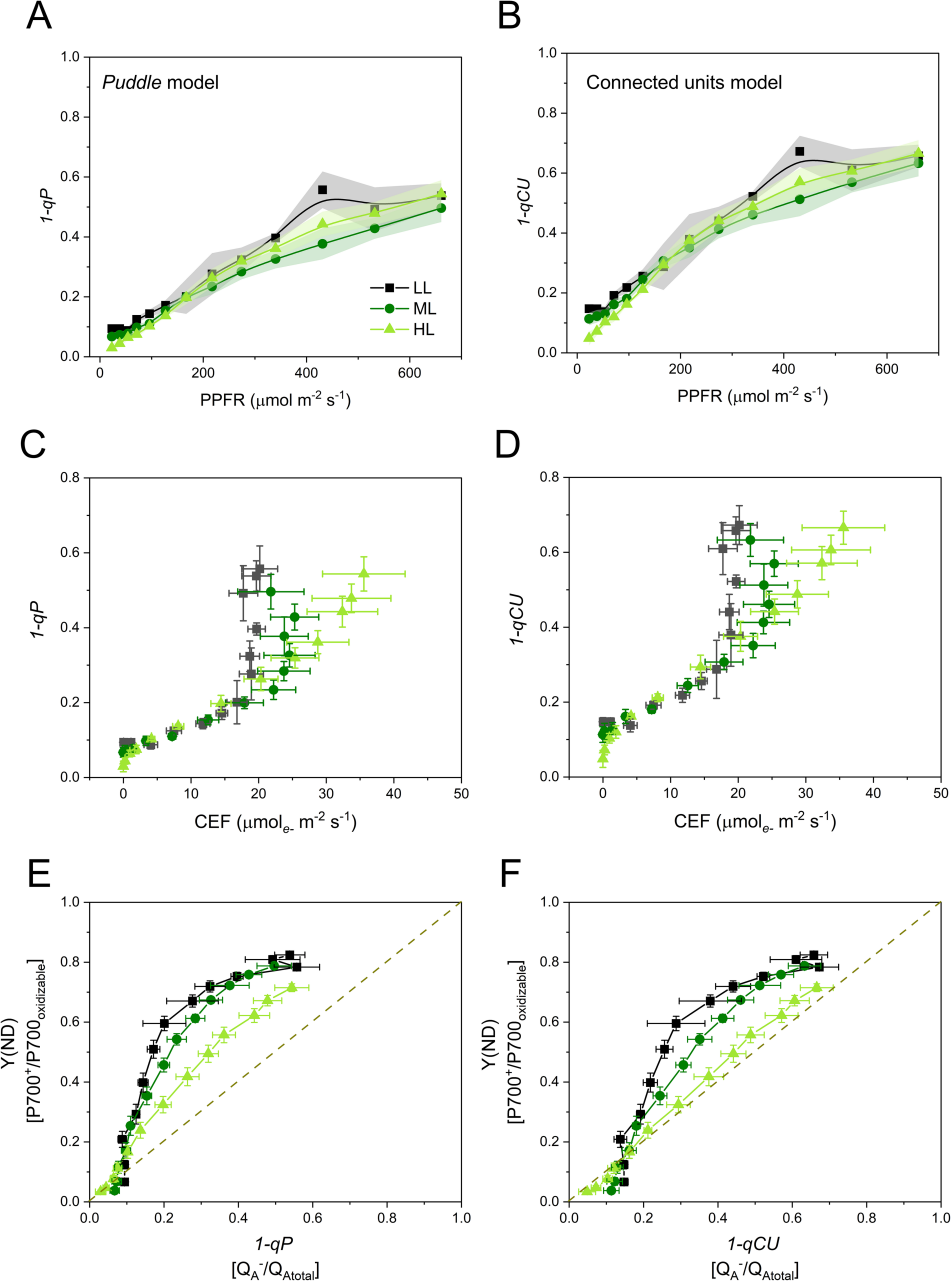
Redox state of the plastoquinone Q_A_ in *Selaginella martensii* plants long‐term acclimated to deep shade (LL), mid‐shade (ML), or high‐light (HL) natural regimes. Calculation was done under two hypotheses, either a *puddle* model of PSII organisation using parameter 1 − *qP* (A, C, E) or a connected PSII units organisation using parameter 1 − *qCU* (B, D, F). (A, B) Light curves. (C, D) Relationship between Q_A_ reduction and cyclic electron flow (CEF). (E, F) Covariation of Q_A_ reduction and P700 oxidation; the expected symmetry is represented by the bisector (dashed line). Average values from 4 to 5 independent plants are shown with SE as a shadowed band (A, B) or error bars (C–F).

While Y(NO) typically indicates the PQ pool reduction state (Tikkanen et al. 2017; Zivcak et al. 2019), its relationship with irradiance and CEF rates in HL plants suggested otherwise for *S. martensii* . Therefore, the Q_A_ redox state was approximated by 1 − *qP*, which increased steadily with the light intensity without any major difference between plant groups, therefore indicating an excellent control of the PQ redox poise (Figure 8A). Moreover, 1 − *qP* increased linearly with CEF rates until saturation of CEF (Figure 8C). A fine‐tuned regulation of electron transport is generally expected to result in a symmetric increase of reduced Q_A_ (1 − *qP*) and oxidized P700 [Y(ND)] because of “bottleneck effect” occurring at the cyt*b*_*6*_ *f* (Cardol et al. 2011; Zivcak, Kalaji, et al. 2014; Zivcak et al. 2015). However, in *S. martensii* , the balance impressively shifted toward the oxidation of the PSI donor‐side in LL plants (Figure 8E). Given this unexpected result, we considered the possibility that 1 − *qP* had underestimated the Q_A_ redox state because it neglects the potential connectivity between PSII units (Kramer et al. 2004; Zivcak, Brestic, et al. 2014). Using the connectivity parameter *p* calculated from the OJIP analysis (Table 1), the Q_A_ redox state was calculated for a connected‐units system using 1 − *qCU*, which, as expected, gave higher estimates than 1 − *qP*, but again without differences between the three plant groups (Figure 8B,D). However, only HL plants featured the anticipated symmetry between PQ reduction and P700 oxidation. Conversely, ML plants, and even more so LL plants, still exhibited a pronounced shortage of electrons at the donor side of PSI (Figure 8F).

## 4 Discussion

The paradigm of the long‐term thylakoid regulation is well‐established, especially, but not exclusively, in angiosperms (Anderson et al. 2012; Bielczynski et al. 2016; Lichtenthaler et al. 2013; Huang et al. 2015; Mathur et al. 2018; Pantaleoni et al. 2009; Shuang et al. 2022; Schumann et al. 2017; Tan et al. 2020; Zivcak, Brestic, et al. 2014). It was excellently summarized by Jan Anderson et al. (2012): compared to shade‐plants, “sun and high‐light plants, being limited in electron transport rather than in light energy capture and conversion, have greater amounts of cyt*b*_*6*_f complexes, ATP synthase and mobile plastoquinone, plastocyanin and ferredoxin, to support greater maximal rates of photosynthesis which saturate at higher irradiance”. From LL to HL regime, at first glance, the functional changes occur in *S. martensii* according to the same direction. However, can one reasonably conclude that in *S. martensii* all aspects conform to current models of membrane photoregulation? Our findings contribute new insights into the specificity of photosynthetic regulation in this lycophyte representative, with regard to its ability to coordinate PSII and PSI activity and achieve high levels of PSI oxidation under contrasting light regimes.

### 4.1 CEF Is Enhanced Upon Long‐Term Acclimation to High Light

Chlorophyll *a* fluorescence and P700 oxidation kinetics indicate downsizing of both PSII and PSI antenna in HL plants (Figure 1D and Table S1), suggesting a lesser investment in light harvesting in sun‐exposed plants (Anderson et al. 2012). However, the LHCII amount is known to be almost invariable in *S. martensii* (Ferroni et al. 2016), and therefore the LHCII excess from the reaction centers can form the population of uncoupled LHCII antennae that we previously reported to steadily increase from LL to HL plants (Colpo et al. 2022). The similar extent of antenna reduction in PSII and PSI can explain the invariant excitation energy distribution between PSII and PSI across the three plant groups. More interesting is the strong preferential excitation of PSI quantified by 1 − d_II_ ~ 0.7 in the light‐acclimated samples, but also confirmed by P700^+^ accumulation kinetics in dark‐acclimated plants (Figures 2 and 6C insert). This finding further strengthens prior reports in *S. martensii* showing that the abundant trimeric LHCII acts as a flexible antenna for both PSII and PSI, particularly through the supramolecular arrangement of PSI‐LHCI, PSII and LHCII into megacomplexes that enhance PSI excitation (Ferroni et al. 2014, 2016, 2018). The excitation reaching PSI can be either used for photochemistry or, in case the reaction center contains P700^+^, be safely dissipated non‐photochemically (Shimakawa and Miyake 2018). In angiosperms, the relative importance of PSI photochemical or non‐photochemical processes depends on the growth light regime: PSI activity is kept at higher levels in high light‐acclimated plants, while low light‐acclimated plants induce more intense PSI oxidation already at relatively low irradiance (Figure 7B; Huang et al. 2015; Schumann et al. 2017; Shuang et al. 2022). These trends are clearly confirmed in *S. martensii* (Figures 3 and 6; Figure S6).

HL *S. martensii* was already known to increase the relative quantity of PSI, cyt*b*_*6*_ *f* and ATP synthase (Ferroni et al. 2016), leading to increased LEF and capacity of ATP synthesis as obvious functional consequences (Figures 4C and 6A,D). The fate of the 2.5‐fold increase in LEF determined by chlorophyll *a* fluorescence can be carboxylation, photorespiration, and alternative electron sinks. It was previously estimated that all of these processes are approximately twice as intense in HL as in LL plants (Ferroni, Brestič, et al. 2021). However, among the several changes from LL to HL plants, the most striking is the threefold rise in PSI concentration, which was known after Ferroni et al. (2016) and accompanied by a parallel decrease in PSII, but also by an impressive increase in NA(P)H dehydrogenase‐like complex (NDH; for effectors of CEF, see Yamori and Shikanai 2016). These changes were strongly suggestive of an upregulation of CEF around PSI, which would also be aligned with expectations based on angiosperms (Miyake et al. 2005; Huang et al. 2015). Fan et al. (2016) reviewed several methods for the quantitative analysis of CEF, evidencing the shortcomings of each of them, and concluded that the best estimates are obtained by subtracting ETR(II) from ETR(I), provided that they are measured from whole leaves under identical conditions. Not surprisingly, the user‐friendly, in vivo simultaneous P700^+^ absorption and chlorophyll *a* fluorescence analysis of Y(PSI) and Y(PSII) enabled by Dual‐PAM technology has become a preferred method for CEF quantification, either indirectly as Y(PSI)/Y(PSII) (e.g., Zivcak et al. 2013) or directly as electron flow (e.g., Huang et al. 2015). Taking into account the excitation partitioning between PSI and PSII in *S. martensii* , the CEF appeared progressively activated according to the light intensity, and also at increasing levels from LL to HL plants (Table 2 and Figure 6F). Despite possible methodological weaknesses (Fan et al. 2016; Furutani et al. 2022), the result is clear and consistent with the commonly accepted physiological significance of CEF (Munekage et al. 2004; Shikanai 2016). The quasi‐linear LEF‐CEF relationship at low LEF levels reflects the need to adjust the ATP:NADPH ratio for the Calvin–Benson–Bassham cycle; accordingly, this phase is more evident in HL plants owing to their higher carboxylation capacity (Figure 6G; Ferroni, Brestič, et al. 2021). Subsequently, CEF increases until it exceeds LEF, and this evidence matches the role of CEF in reducing the excitation pressure inside PSII by facilitating the NPQ induction (Joliot and Finazzi 2010), but especially in preserving PSI integrity by alleviating the electron pressure (Munekage et al. 2004). PSI oxidation under high light can be additionally promoted in *S. martensii* by other downstream electron‐consuming pathways besides CEF, particularly FLVs‐mediated O_2_ photoreduction (Ilík et al. 2017). The synergistic and additive photoprotective action of CEF and pseudo‐cyclic electron routes has been emerging with clarity in the model non‐vascular species *Physcomitrium patens*, evidencing an only partially functional overlap of NDH‐ and PGR5/PGRL1‐dependent CEF with FLV‐mediated O_2_ photoreduction, as well as their long‐term modulation (Storti et al. 2019; Beraldo et al. 2025). A similar synergistic action is very probable also in lycophytes, which, despite being true vascular plants, share some photosynthetic features with mosses (e.g., LHCB6 protein phosphorylation, Gerotto et al. 2019; Ferroni et al. 2014). Future research in lycophytes could especially focus on the alternative sink represented by FLVs, which seems positively modulated from LL to HL plants based on P700 re‐oxidation during the SP (Figure S3).

### 4.2 Long‐Term Modulation of Electron Transport Acts on the Capacity and Mobility of the Electron Carriers Related to Thylakoid Shrinkage

Not all parameters display a gradient from LL to HL plants; particularly, OJIP‐derived electron transporter pools show little difference: PQ pool size remains nearly constant, and the end acceptor pool increases from LL to ML but levels off in HL plants (Table 1). These trends do not show evident parallels with the steady increase in the relative PSI amount, confirming that the latter can be only weakly related to the I‐P phase amplitude (Zivcak et al. 2015; Ferroni et al. 2022). The similarity of the OJIP transients among LL, ML, and HL plants also emerges when fluorescence is recorded via amplitude modulation; nonetheless, the simultaneous recording of P700 oxidation kinetics again reveals a clear gradient in the rate of P700^+^ re‐reduction (Figure 2 and Table S1). This inconsistency can be addressed by considering not only the availability but also the mobility of electron transporters. The photosynthetic membranes of *S. martensii* are characterized by exceptionally long appressed disks and protein overcrowding due to the very extensive LHCII moiety (Ferroni et al. 2016; Colpo et al. 2023). Both conditions significantly restrict the electron flow, particularly the diffusion of the long‐range electron carrier plastocyanin (Höhner et al. 2020). In LL and ML plants, the light‐acclimated thylakoids are strongly shrunken: a lumen width of 6.5 nm is barely enough for plastocyanin to diffuse (4–5 nm in diameter; Kirchhoff et al. 2011) and, accordingly, the re‐reduction of P700^+^ is particularly slow (Figures 2 and 5; Table S1; Colpo et al. 2023). Wójtowicz et al. (2025) have demonstrated the complexity of the trade‐off between thylakoid shrinkage and expansion upon illumination, underlying how the former enhances the CEF/LEF ratio by hindering the mobility of plastocyanin. This inference is strongly supported in *S. martensii* LL plants: with their minimal PSI content, they can develop a limited absolute CEF (~20 μmol electrons m^−2^ s^−1^), yet CEF extensively surpasses LEF (Table 2). Conversely, HL plants achieve a CEF/LEF ratio near one within more expanded thylakoids, which ensure a less constrained diffusion of plastocyanin from PSII to PSI, doubling the P700^+^ re‐reduction rate. Other functional parallels of more dilated thylakoids in HL than in LL plants are smaller *pmf* and faster stabilization of Y(NPQ) during light induction, which can be easily associated with more active ATP synthase and more diluted protons in the lumen (Figures 3C and 4B,C). In summary, from LL to ML, the electron flow is improved by more capacitive electron carrier pools per PSII‐PSI chain unit, while it is improved by increasing the efficiency of their diffusion from ML to HL. Interestingly, the tendency for HL plants to exhibit slightly higher levels of PSI overreduction may be related to the electron transporters pool size, which remains as small as in ML plants (Figure 3F and Figure S6). It is also conceivable that in HL plants, the upregulation of FLVs may help control the electron poise in the absence of large changes in pools of electron transporters.

### 4.3 PSI Is Kept Under a Persistent Shortage of Electrons Especially in Shade‐Grown Plants

Unexpectedly, under all light regimes in *S. martensii* , the covariation of 1 − *qP* and Y(ND) suggests that the fraction of oxidised P700 exceeds that of reduced Q_A_ (Figure 8E). On the one hand, although LEF is saturated at 50 to 125 μmol electrons m^−2^ s^−1^ depending on the growth light regime, the PQ pool reduction is far from being saturated even at 660 μmol electrons m^−2^ s^−1^. On the other hand, PSI is kept under a persistent shortage of electrons, which is against a common principle of photosynthetic control at the cyt*b*_*6*_ *f*, that is, that the rates of PQ reduction and PSI oxidation should be equivalent to each other to sustain the LEF, leading to equal rates of electron influx into and efflux from PSI (Zivcak, Kalaji, et al. 2014; Zivcak et al. 2015; Kono and Terashima 2016).

A seemingly excessive fraction of oxidised PSI in all plant groups could raise doubts about the reliability of 1 − *qP* as a proxy for the reduction state of Q_A_. Particularly, the regulation of the LEF can be influenced by the connectivity between PSII units, which could cause excitation imbalances between PSII and PSI (Johnson and Berry 2021). PSII connectivity refers to the transfer of excitation energy from a closed to a nearby open PSII, as allowed by the LHCII antenna lake embedding the PSII units (Joliot and Joliot 1964; for a comprehensive review, see Stirbet 2013). However, the relevance of excitonic connectivity between PSII units remains controversial, with some researchers disputing its existence or measurability from fluorescence transients (Vredenberg 2008; Oja and Laisk 2020; Garab et al. 2023). Nevertheless, the assessment of PSII connectivity parameters can offer valuable insights into thylakoid photoregulation (Brestic et al. 2012; Ceusters et al. 2019; Wood and Johnson 2020; Shi et al. 2024). In barley, a lower PSII connectivity in shade than in sun leaves results in fewer chances to use excitons for PSII photochemistry and, consequently, helps keep the excitation pressure under control under high light, facilitating PSII photoprotection (Zivcak, Brestic, et al. 2014). We could not differentiate the three *S. martensii* groups based on PSII connectivity, which rather appears to be a relatively invariable property across LL, ML and HL plants (Table 1). Such an invariability can depend on the very large excess of free LHCII trimers compared to PSII under the three conditions (Ferroni et al. 2016), which may allow the maintenance of a constant distance between PSII units and/or a similar protein density in grana cores (see Haferkamp et al. 2010). Conceivably, a certain probability of exciton sharing by neighboring PSII reaction centers increases the efficiency of light energy use for charge separation. Accordingly, in the model by Kramer et al. (2004), PSII connectivity is used to derive the parameter *qCU* (and *qL* in an almost pure “lake model”), whose complement to unity would be a better estimator of the reduction state of Q_A_ than 1 − *qP*. The use of 1 − *qCU* is based on strong assumptions, particularly that PSII connectivity truly exists, can be estimated from OJIP transients, and influences the redox state of the PQ pool. When applied to the case of *S. martensii* , the 1 − *qCU* versus Y(ND) covariation indicates that the over‐accumulation of oxidized PSI is specific to the long‐term shade acclimation, whereas HL plants now conform to the general rule of balanced Q_A_ reduction and P700 oxidation (Figure 8F), which is in agreement with their more intense LEF and higher strength of electron sinks.

## 5 Conclusion

From deep shade to high light, *S. martensii* increases LEF and CEF, meeting expectations related to the gain in carboxylation capacity and higher PSI/PSII ratio. These changes occur in the context of a downsizing of the functional antenna of both photosystems without significant changes in PSII connectivity. The long‐term modulation of photosynthetic electron transport exhibits interesting differences compared to current models of membrane photoregulation:

- Under any light regime, *S. martensii* excites preferentially PSI, even when it is present in minute amounts in shade plants;
- The pools of electron transporters do not increase linearly with the electron fluxes; instead, the enhancement of ETRs depends on unique combinations of capacity and mobility of the electron carriers, the latter determined by a level of thylakoid ultrastructural control;
- Especially under deep shade, the prompt accumulation of oxidised PSI reaches outstanding levels, exceeding the rate of Q_A_ reduction.

Under any light regime, *S. martensii* appears well protected against the overreduction of PSI acceptor side—and consequently PSI photodamage—thanks to a prominent PSI oxidation. Further comparative studies may determine if exceptionally high Y(ND) values relative to reduced Q_A_ are unique to lycophytes or also found in phylogenetically distant deep‐shade species.

## Author Contributions

Lorenzo Ferroni conceived and planned the experiments. Lorenzo Ferroni performed most of the experiments and data elaborations. Stefania Simonetto performed fast chlorophyll fluorescence experiments. Andrea Colpo validated the chlorophyll fluorescence results. Lorenzo Ferroni, Marek Živčak, Marian Brestič, Costanza Baldisserotto, and Simonetta Pancaldi analyzed the data and interpreted the results. Marian Brestič provided resources to conduct the research. Lorenzo Ferroni wrote the manuscript. All authors edited the manuscript.

## Funding

This research was funded by European Commission (EPPN2020‐OPVaI‐VA—ITMS 313010T813; granted to M.B.), Science Grant Agency of The Ministry of Education, Science, Research and Sport of the Slovak Republic—VEGA 1‐0425‐23 (M.Ž.), the Slovak Academic Information Agency (scholarships granted to L.F. and A.C.), and the University of Ferrara (Fondo per l'Incentivazione alla Ricerca Dipartimentale—FIRD 2023—granted to L.F.).

## Supporting information


**Table S1:** Parameters derived from fast P700 oxidation kinetics of *Selaginella martensii* plants long‐term acclimated to deep shade (LL) mid shade (ML) or high light (HL) natural regimes. Values (means with SE) were obtained upon induction with a 0.6 s saturation pulse of 3000 μmol m^−2^ s^−1^ (*N* = 3–6; see Figure 2 in the main text). P700^+^ accumulation rate was calculated as the initial signal rise (0–1.2 ms) and normalized on P_M_. P700^+^ accumulation rate was calculated as the slope within the 100–200 ms time interval and normalized on P_M_. Chlorophyll content was determined in acetonic extracts. Data were treated with ANOVA and different letters indicate a significant difference with *p* < 0.05 as resulting from post hoc Tukey's test.
**Figure S1:** Protocol of irradiance variation during Dual‐PAM analysis of chlorophyll *a* fluorescence and P700 redox state.
**Figure S2:** Graphical analysis of the sigmoidal character within 300 μs in fast chlorophyll *a* fluorescence transients recorded from *Selaginella martensii* long‐term acclimated to deep shade (LL) mid shade (ML) or high light (HL) natural regimes. *W* represents the experimental fluorescence curve double normalized between 20 (O step) and 2000 μs (J step); *W*_*E*_ represents the purely exponential fluorescence rise, assuming that *W* and *W*_*E*_ converge at 300 μs. The sigmoidal character assigned to PSII connectivity is visualized as the difference Δ*W* between *W*_*E*_ and *W*. Positive Δ*W* peaks in all plant groups in the range between 100 and 150 μs (L band), without evident differences among plants, which, according to Strasser and Stirbet (2001), indicates a similar level of PSII exciton connectivity in all conditions.
**Figure S3:** Representative fast kinetics of the relative amount of P700^+^ in *Selaginella martensii* plants long‐term acclimated to deep shade (LL) mid shade (ML) or high light (HL) natural regimes. After 30 min dark‐acclimation, the P700^+^ signal was recorded using the saturation pulse method (see in main text 2.3). For easier comparison, the signal was double‐normalized and linear time scale was used. The relatively slow rise after 400 ms is attributed to the O_2_ photoreduction allowed by electron flow from P700 to downstream flavodiiron proteins (Ilík et al., *New Phytologist*, 214(3), 2017, 967–972).
**Figure S4:** Representative rough traces of chlorophyll *a* fluorescence and P700^+^ absorption obtained during the exposure to increasing irradiance in light‐acclimated *Selaginella martensii* grown under deep shade (LL) mid shade (ML) or high light (HL). The measuring routine is described in the main text Section 2.3 and represented schematically in Figure S1. P700^+^ are shown before automatic slope correction by Dual‐PAM software. Chlorophyll *a* fluorescence is shown with reference minimum *F*_0_ and maximum *F*_0_ values in the dark‐acclimated state. The traces are shown after a 30‐point averaging.
**Figure S5:** Transmission electron micrographs of chloroplasts in the upper epidermal cells of *Selaginella martensii* plants long‐term acclimated to deep shade (A, B) mid shade (C, D) or high light (E, F) natural regimes. The plants were exposed to 200 μmol m^−2^ s^−1^ for 1 h before fixation. In all cases, a single large chloroplast with an abundant thylakoid system is present in each cell. (A, B) In deep‐shade plants, the organelle contains a homogeneous pseudolamellar thylakoid system without clear differentiation of individual grana. (C) In mid‐shade plants, the organelle is a bizonoplast with a slight upper concavity, and features a lamellar thylakoid region at the top and a granal organization beneath; (D) in the detail, the grana appear irregular in shape. (E) Under high light regime, the bizonoplast concavity is more marked and the thylakoid zonation remains evident; (F) the granal organization of the thylakoid system is similar to that in mid‐shade plants.
**Figure S6:** Light curves of non‐photochemical quantum yields in *Selaginella martensii* plants long‐term acclimated to deep shade (LL) mid shade (ML) or high light (HL) natural regimes. Quantum yield of regulatory non‐photochemical energy dissipation Y(NPQ), non‐regulatory energy dissipation Y(NO), non‐photochemical energy dissipation in donor‐side limited PSI Y(ND), non‐photochemical energy dissipation in acceptor‐side limited PSI Y(NA). Average values are shown with SE, represented as shadowed bands, from 4 to 5 independent plants.

## Data Availability

The data that support the findings of this study are available from the corresponding author upon reasonable request.
