# Supplementary material for: Coordinated Activity of Photosystem I and Photosystem II in Selaginella martensii (Lycopodiophyta) Across Light Gradients
Source: Physiol Plant. 2026 Apr 4;178(2):e70857. doi: 10.1111/ppl.70857 (PMC13050137; doi:10.1111/ppl.70857)
Supplement: Supplementary file 1 — Table S1: Parameters derived from fast P700 oxidation kinetics of Selaginella martensii plants long‐term acclimated to deep shade (LL) mid shade (ML) or high light (HL) natural regimes. Values (means with SE) were obtained upon induction with a 0.6 s saturation pulse of 3000 μmol m−2 s−1 (N = 3–6; see Figure 2 in the main text). P700+ accumulation rate was calculated as the initial signal rise (0–1.2 ms) and normalized on PM. P700+ accumulation rate was calculated as the slope within the 100–200 ms time interval and normalized on PM. Chlorophyll content was determined in acetonic extracts. Data were treated with ANOVA and different letters indicate a significant difference with p < 0.05 as resulting from post hoc Tukey's test. Figure S1: Protocol of irradiance variation during Dual‐PAM analysis of chlorophyll a fluorescence and P700 redox state. Figure S2: Graphical analysis of the sigmoidal character within 300 μs in fast chlorophyll a fluorescence transients recorded from Selaginella martensii long‐term acclimated to deep shade (LL) mid shade (ML) or high light (HL) natural regimes. W represents the experimental fluorescence curve double normalized between 20 (O step) and 2000 μs (J step); W E represents the purely exponential fluorescence rise, assuming that W and W E converge at 300 μs. The sigmoidal character assigned to PSII connectivity is visualized as the difference ΔW between W E and W. Positive ΔW peaks in all plant groups in the range between 100 and 150 μs (L band), without evident differences among plants, which, according to Strasser and Stirbet (2001), indicates a similar level of PSII exciton connectivity in all conditions. Figure S3: Representative fast kinetics of the relative amount of P700+ in Selaginella martensii plants long‐term acclimated to deep shade (LL) mid shade (ML) or high light (HL) natural regimes. After 30 min dark‐acclimation, the P700+ signal was recorded using the saturation pulse method (see in main text 2.3). For easier compar [file PPL-178-e70857-s001.pdf]

SUPPLEMENTALS

Coordinated activity of photosystem I and photosystem II in *Selaginella martensii* (Lycopodiophyta) across light gradients

Lorenzo Ferroni[1,2,*], Marek Živčak[2], Andrea Colpo[1,2], Stefania Simonetto[1], Costanza Baldisserotto[1], Simonetta Pancaldi[1], Marian Brestič[2]

[1] Department of Environmental and Prevention Sciences, University of Ferrara, Corso Ercole I d'Este 32, 44121 Ferrara, Italy.

[2] Institute of Plant and Environmental Sciences, Faculty of Agrobiology and Food Resources, Slovak University of Agriculture, Trieda A. Hlinku 2, 94976 Nitra, Slovakia.

[*]Corresponding author, e-mail: lorenzo.ferroni@unife.it

Supporting information

Table S1. Parameters derived from fast P700 oxidation kinetics.

Figure S1. Protocol of irradiance variation during Dual-PAM analysis of chlorophyll *a* fluorescence and P700 redox state.

Figure S2. Graphical analysis of the sigmoidal character within 300 μs in fast chlorophyll *a* fluorescence transients.

Figure S3. Representative fast kinetics of the relative amount of $P700^+$ in a linear time scale.

Figure S4. Representative rough traces of chlorophyll *a* fluorescence and $P700^+$ absorption obtained during the exposure to increasing irradiance.

Figure S5. Transmission electron micrographs of chloroplasts in the upper epidermal cells.

Figure S6. Light curves of non-photochemical quantum yields.

Table S1 Parameters derived from fast P700 oxidation kinetics of *Selaginella martensii* plants long-term acclimated to deep shade (LL) mid shade (ML) or high light (HL) natural regimes. Values (means with SE) were obtained upon induction with a 0.6 s saturation pulse of 3000 µmol m^{-2} s^{-1} (*N*=3-6; see Figure 2 in the main text). $P700^{+}$ accumulation rate was calculated as the initial signal rise (0-1.2 ms) and normalized on P_M. $P700^{+}$ accumulation rate was calculated as the slope within the 100-200 ms time interval and normalized on P_M. Chlorophyll content was determined in acetonic extracts. Data were treated with ANOVA and different letters indicate a significant difference with $p<0.05$ as resulting from post-hoc Tukey's test.

**Parameter**	**LL**	**ML**	**HL**
P_M	0.592 ± 0.039^{b}	0.927 ± 0.069^{a}	1.012 ± 0.054^{a}
Chlorophyll (nmol mg^{-1})	1.87 ± 0.12^{a}	1.62 ± 0.19^{a}	1.01 ± 0.10^{b}
P_M/Chlorophyll	0.32	0.57	1.00
$P700^{+}$ accumulation rate (ms^{-1})	0.117 ± 0.014^{a}	0.096 ± 0.007^{ab}	0.075 ± 0.003^{b}
$P700^{+}$ re-reduction rate (s^{-1})	0.775 ± 0.203^{b}	1.299 ± 0.171^{ab}	1.730 ± 0.169^{a}

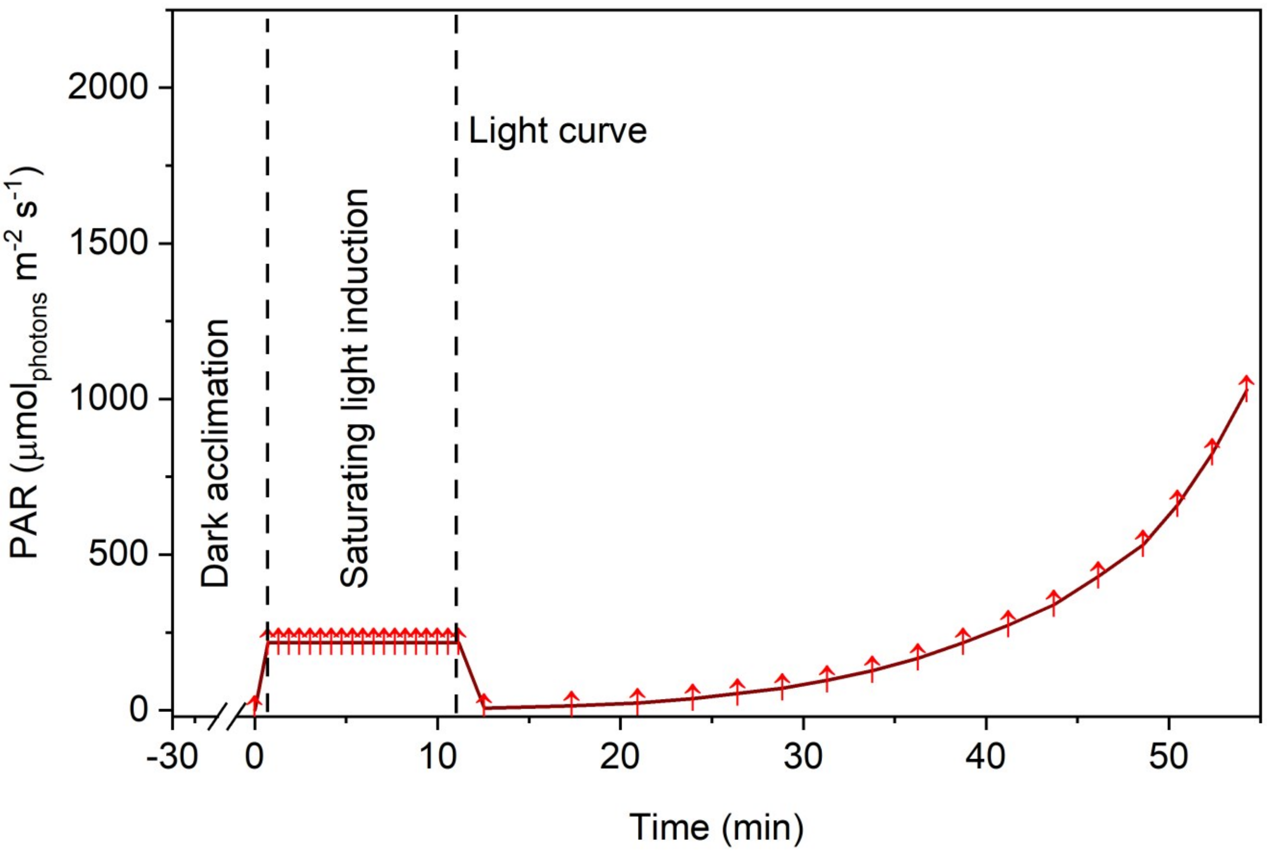

Figure S1. Protocol of irradiance variation during Dual-PAM analysis of chlorophyll *a* fluorescence and P700 redox state.

After the initial dark acclimation for 30 min, the samples were exposed to a sequence of photosynthetically active radiation (PAR) at variable intensity. The time points at which a saturation pulse was applied are shown with arrows. After determination of the dark-acclimated parameters, a (quasi)steady state of photosynthesis was induced at saturating actinic light (217 µmol m^{-2} s^{-1}). Subsequently, a light curve was recorded exposing the sample to increasing irradiance from 7 to 1089 µmol m^{-2} s^{-1}. During the first 10 min after the end of the induction phase, photosynthesis was acclimated to low irradiance to determine the distribution of excitation energy between PSII and PSI assuming negligible CEF (see main text 2.4).

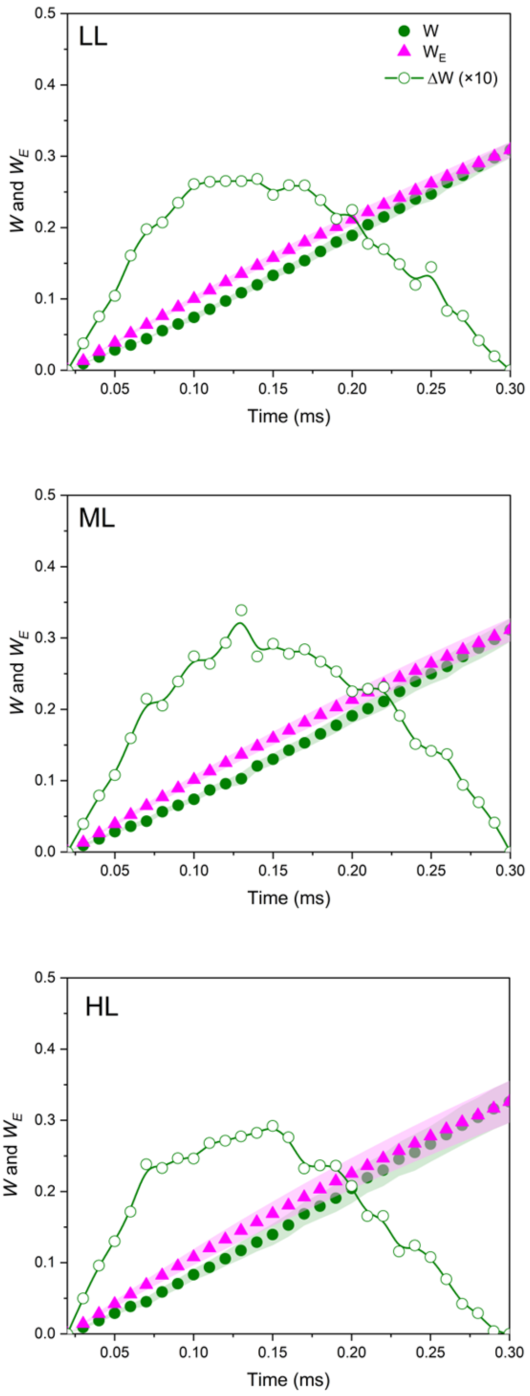

Figure S2. Graphical analysis of the sigmoidal character within 300 µs in fast chlorophyll *a* fluorescence transients recorded from *Selaginella martensii* long-term acclimated to deep shade (LL) mid shade (ML) or high light (HL) natural regimes. W represents the experimental fluorescence curve double normalized between 20 (O step) and 2000 µs (J step); W_E represents the purely exponential fluorescence rise, assuming that W and W_E converge at 300 µs. The sigmoidal character assigned to PSII connectivity is visualized as the difference ΔW between W_E and W. Positive ΔW peaks in all plant groups in the range between 100 and 150 µs (L band), without evident differences among plants, which, according to Strasser and Stirbet (2001), indicates a similar level of PSII exciton connectivity in all conditions.

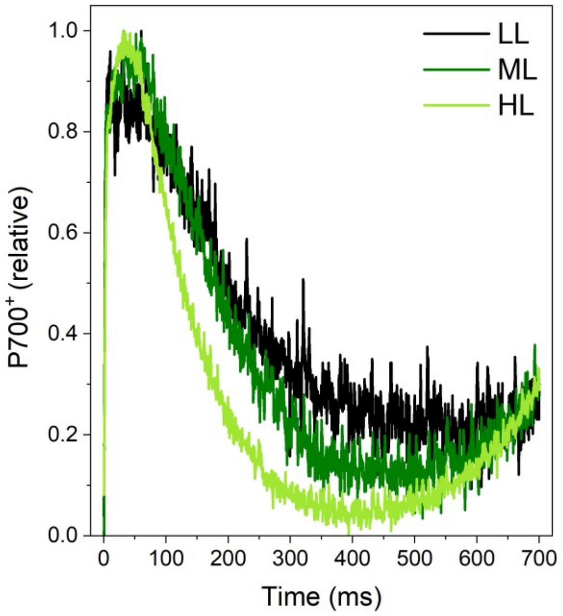

Figure S3. Representative fast kinetics of the relative amount of $P700^+$ in *Selaginella martensii* plants long-term acclimated to deep shade (LL) mid shade (ML) or high light (HL) natural regimes. After 30 min dark-acclimation, the $P700^+$ signal was recorded using the saturation pulse method (see in main text 2.3). For easier comparison, the signal was double-normalized and linear time scale was used. The relatively slow rise after 400 ms is attributed to the O_2 photoreduction allowed by electron flow from P700 to downstream flavodiiron proteins (Ilík et al., *New Phytologist*, 214(3), 2017, 967-972).

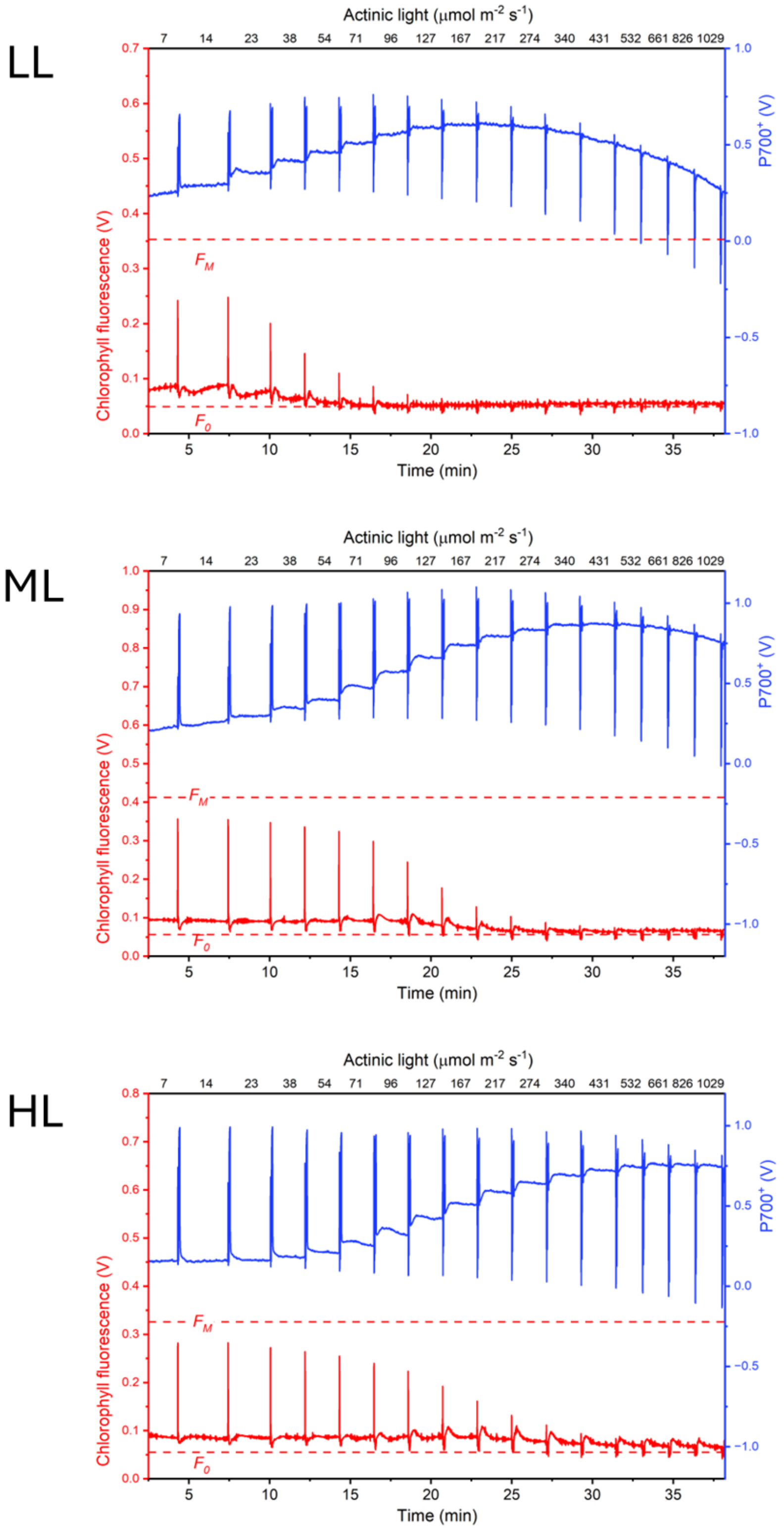

Figure S4. Representative rough traces of chlorophyll *a* fluorescence and P700^{+} absorption obtained during the exposure to increasing irradiance in light-acclimated *Selaginella martensii* grown under deep shade (LL) mid shade (ML) or high light (HL). The measuring routine is described in the main text section 2.3 and represented schematically in Fig. S1. P700^{+} are shown before automatic slope correction by Dual-PAM software. Chlorophyll *a* fluorescence is shown with reference minimum F_0 and maximum F_0 values in the dark-acclimated state. The traces are shown after a 30-point averaging.

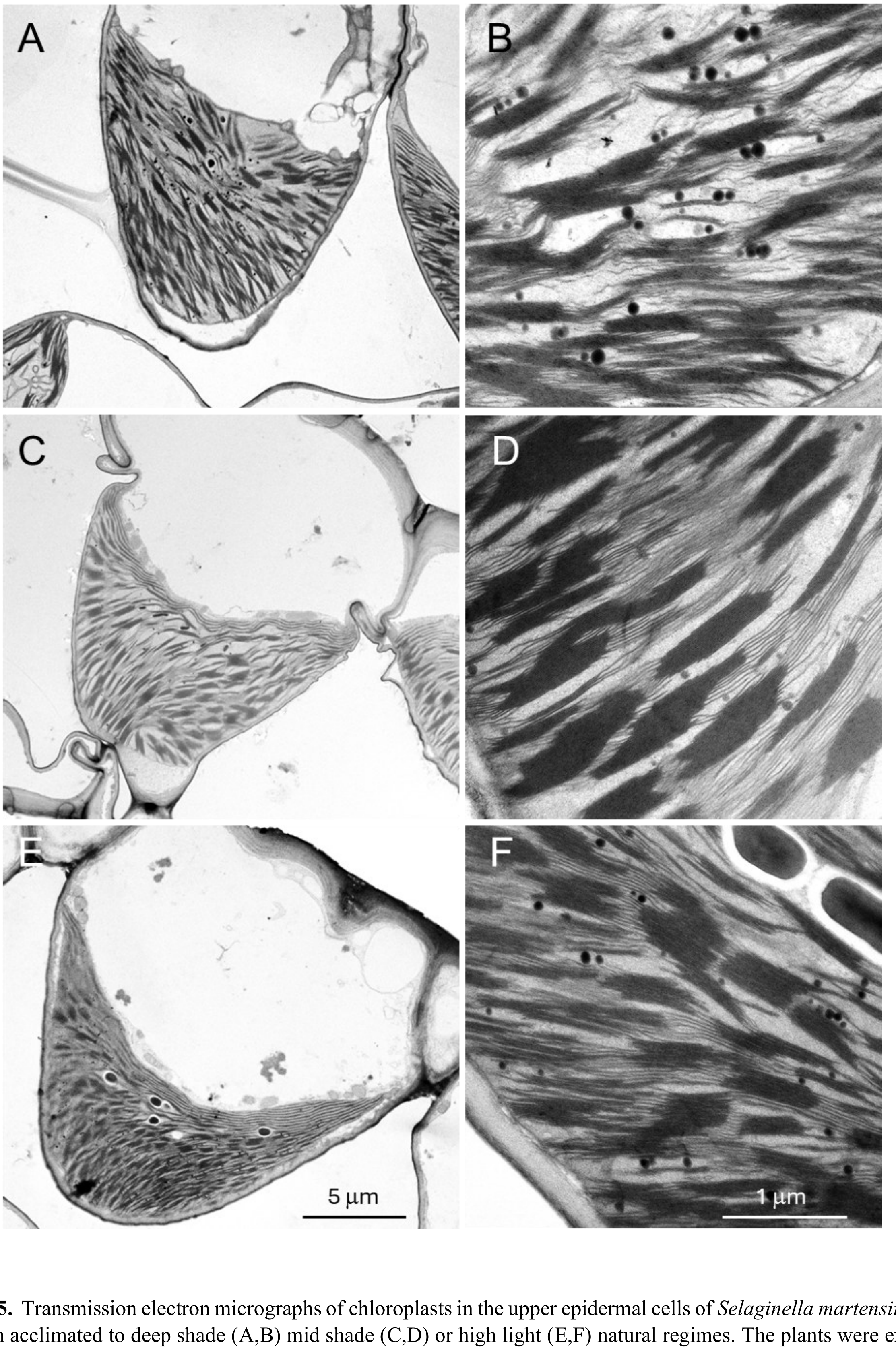

Figure S5. Transmission electron micrographs of chloroplasts in the upper epidermal cells of *Selaginella martensii* plants long-term acclimated to deep shade (A,B) mid shade (C,D) or high light (E,F) natural regimes. The plants were exposed to 200 µmol m^{-2} s^{-1} for 1 h before fixation. In all cases, a single large chloroplast with an abundant thylakoid system is present in each cell. (A, B) In deep-shade plants, the organelle contains a homogeneous pseudolamellar thylakoid system without clear differentiation of individual grana. (C) In mid-shade plants, the organelle is a bizonoplast with a slight upper concavity, and features a lamellar thylakoid region at the top and a granal organization beneath; (D) in the detail, the grana appear irregular in shape. (E) Under high light regime, the bizonoplast concavity is more marked and the thylakoid zonation remains evident; (F) the granal organization of the thylakoid system is similar to that in mid-shade plants.

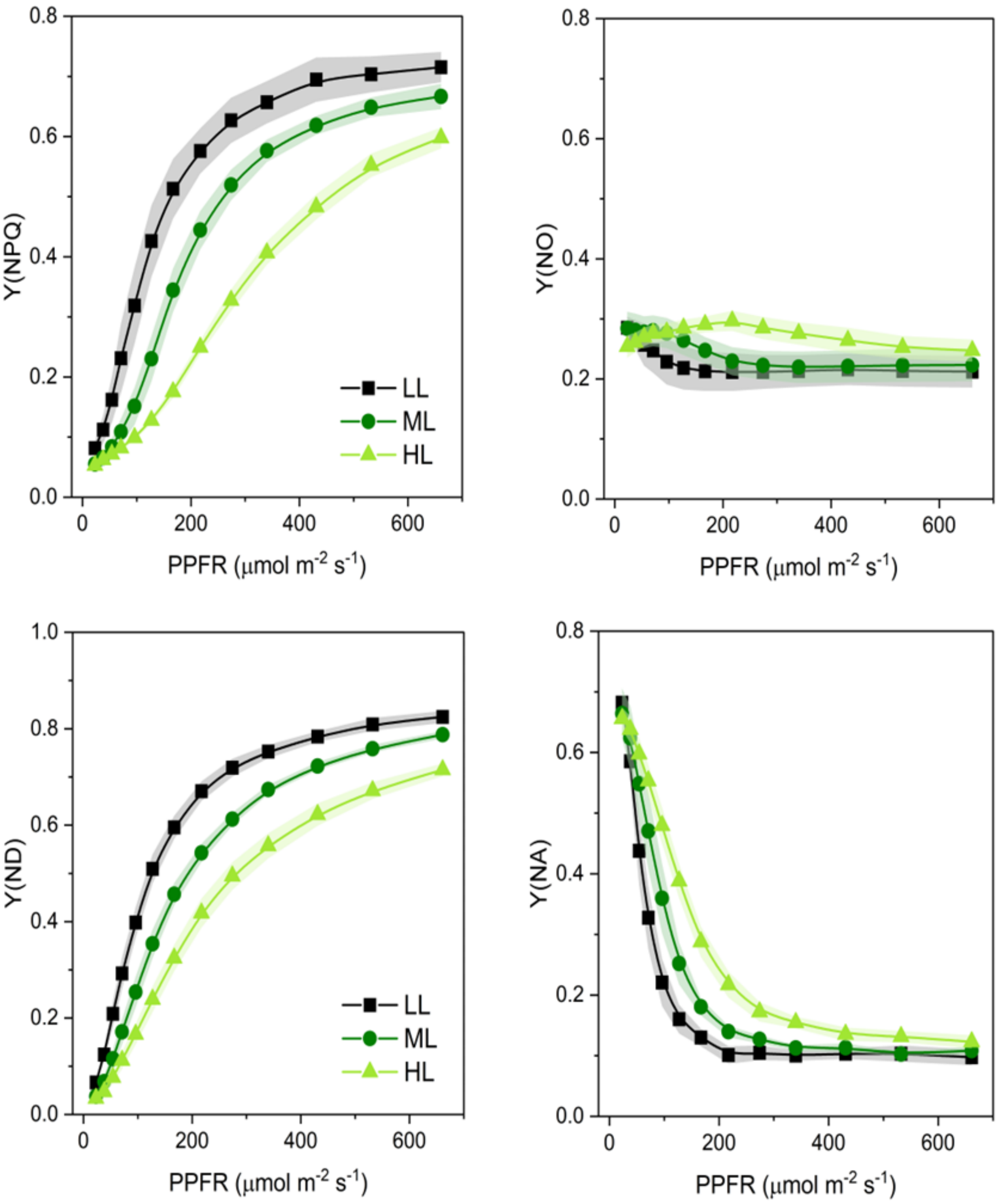

Figure S6. Light curves of non-photochemical quantum yields in *Selaginella martensii* plants long-term acclimated to deep shade (LL) mid shade (ML) or high light (HL) natural regimes. Quantum yield of regulatory non-photochemical energy dissipation Y(NPQ), non-regulatory energy dissipation Y(NO), non-photochemical energy dissipation in donor-side limited PSI Y(ND), non-photochemical energy dissipation in acceptor-side limited PSI Y(NA). Average values are shown with SE, represented as shadowed bands, from 4-5 independent plants.
